# Supplementary material for: African inland wetland area on the rise during the 21st century
Source: Nat Commun. 2026 Mar 6;17:3600. doi: 10.1038/s41467-026-70480-6 (PMC13096438; doi:10.1038/s41467-026-70480-6)
Supplement: Supplementary file 1 — Supplementary Information [file 41467_2026_70480_MOESM1_ESM.pdf]

Supplementary Information for
African inland wetland area on the rise during the 21st century

Anzhen Li[1], Shengbo Chen[1*], Kaishan Song[2], Yuanzhi Zhang[3,4,**], Zongming Wang[2], Dehua Mao[2]

[1] College of Geo-Exploration Science and Technology, Jilin University, Changchun 130026, China
[2] Northeast Institute of Geography and Agroecology, Chinese Academy of Sciences, Changchun 130102, China
[3] School of Marine Sciences, Nanjing University of Information Science and Technology, Nanjing 210044, China
[4] Department of Architecture and Civil Engineering, Faculty of Engineering, City University of Hong Kong, Hong Kong 999777

[*] and [**] are correspondence to Shengbo Chen (chensb@jlu.edu.cn) and Yuanzhi Zhang (yzhang209@nuist.edu.cn)

Contents of this file

Supplementary Methods and Discussions 1 to 10

Supplementary Tables 1 to 6

Supplementary Figures 1 to 27

Supplementary Methods and Discussions

1. African wetland maps

Wetland mapping methods can be broadly divided into two categories: field-based methods and remote-sensing methods[1]. Unlike traditional methods, wetland mapping using remote-sensing methods has proven to be the most efficient and cost-effective method[2]. Relying on the continuous development of remote-sensing technology, previous studies have proposed several wetland thematic maps, including the global wetland type map by synthesizing the best sources of all available maps by Lehner and Doll in 2004 [3]; a global surface water map at 30 m resolution using Landsat imagery by Pekel et al. in 2016[4]; and an tropical and subtropical wetlands map based on expert systems by Gumbricht et al. in 2017[5]. There were also some wetland maps that did not distinguish between types, such as GIEMS[6], WAD2M[7], etc.

The problem with these wetland thematic maps, however, is that they either differentiate between wetlands as broad categories without detailed wetland category differentiation[8,9]; or compiled datasets resulting from the compilation of historical data[3]; or they depict only a particular type of wetland[10] ,for example, mangroves[11,12], salt marshes[13] or tidal flats[14]. None of these provide systematic and accurate spatial information on wetlands in Africa. There were also global-scale land cover maps produced by remote-sensing datasets[15-17], such as GLC30[18], which generally included the category of wetlands. However, due to the limitation of product use, the remote-sensing classification features of wetlands could not be fully considered when producing such products, so the obtained wetland results often had a large deviation. In Africa, most African wetland maps were concentrated in national or local areas, such as in South Africa[19], Uganda[20], Congo Basin[21], etc. There was no uniform wetland classification scheme and no consistent results that could be compared with each other.

The lack of African wetland maps has also led to a lack of understanding of the changes in the area of African wetlands. Although many studies have been published on wetland change involving the African region, for example, the impact of sea level

rise on coastal wetlands in 2018[22] ; the future simulation study of Ramsar wetland in 2020[23] ; research on global wetland loss and transformation in 2023[24]. However most of these papers were analyses of global wetlands that took wetlands as a broad category and rely on modeling calculations. There was still no relevant research on the classification, mapping and further change analysis of different types of wetland area changes in African wetlands based on Landsat long time series images.

2. Climate change in Africa

Climate change referred to the long-term transformation or alteration of climate at a specific location, region, or globally, resulting from either natural variations or human activities. Africa is the second most populous continent in the world, impacted by climate change while having low adaptive capacity, making it one of regions the most vulnerable to climate change. Particularly, Africa's agricultural sector, which employs over 60% of the population, was highly sensitive to changes in temperature and precipitation. Climate change (e.g., the increased frequency of extreme weather events) was expected to have socioeconomic impacts on Africa. Future climate data from the latest Phase 6 of the Coupled Model Intercomparison Project (CMIP6) had become available for analysis[25]. The CMIP6 comprised multiple climate models, incorporating simulation results from the world's leading climate research institutions. This multi-model ensemble approach allowed researchers to assess inter-model uncertainties and enhance the reliability of predictions through integrated analysis[26].

Some studies indicated that the CMIP6 predicted an increase in precipitation and a rise in temperature across most of Africa[27-30]. In contrast, Southern Africa and Madagascar were projected to become drier in the future[31]. According to the multi-model median, the frequency of extremely wet years was expected to increase fivefold between 2050 and 2100 in most parts of Africa, excluding Southern Africa and Madagascar[29]. Specifically, in the Sahel region, research found that the past two decades (1991-2010) were characterized by a slight wetting trend, with a reduction in the geographic extent affected by drought, while the annual average precipitation in the

21st century was projected to increase[32].

In this study, we also used the CMIP6 data of temperature, precipitation, and soil moisture to evaluate future climate change in Africa. Our conclusions were largely consistent with previous studies. As shown in Supplementary Figure 14 and 15, temperature, precipitation, and soil moisture were projected to increase across most of Africa during the 21st century. The primary areas of temperature increase were concentrated in the regions north of the Sahara (such as Morocco and Algeria) and in Southern Africa (including South Africa, Botswana, and Angola). The changes in precipitation displayed a marked north-south contrast across Africa. The Congo Basin experienced a decrease in precipitation, while the areas in Southern Africa where precipitation decreased corresponded closely with regions of rising temperatures. Future changes in soil moisture were expected to manifest as increases in the Sahel and the Horn of Africa, with decreases in Central and Southern Africa.

3. Wetland area time series

This study produced a total of 10 wetland maps from 1984 to 2021. In these 10 wetland maps, excluding the three maps after 2013, the remaining seven maps had more or less incomplete image coverage (Supplementary Fig. 16). The number of early Landsat images was limited, and even if a time span of 7-years and 5-years was used (1984-1990, 1991-1995, and 1996-2000), this issue could not be improved. However, the missing areas were mainly concentrated in the Sahara Desert area, which basically lacks wetland distribution; therefore, we determined that the issue would not affect the wetland area statistics and subsequent analysis. However, during the period from 1991 to 1995 (the middle year 1993), the issue of missing Landsat images was particularly serious and the missing area involved Central Africa. As a result, our calculation of the area of all categories of wetlands was abnormally low. Therefore, the 1993 wetland results were discarded in the follow-up analysis, and only the results of the remaining nine periods were used for research.

We compared our results with GIEMS-MC, GWL-FCS30, and WAD2M

(Supplementary Figs. 22 – 24). The comparisons for the post-2000 period yield conclusions that can be regarded as robust. Both GIEMS-MC and the 30-m GWL-FCS30 clearly indicate an upward trend in African wetland area. GWL-FCS30, released in 2024, is the most recent global 30-m time-series wetland dynamics dataset and covers 2000 – 2022; its record of interannual variability in African wetlands is broadly consistent with our findings. By contrast, WAD2M shows no clear upward or downward trend and differs substantially from the other two datasets. The GIEMS-MC dataset spans 1992 – 2020 and has a moderate positive linear correlation with our time series (Pearson r = 0.421, p = 0.013). The largest discrepancies between GIEMS-MC and our results are concentrated before 2000: GIEMS-MC exhibits only a slight decline in trend, whereas our results show a more pronounced decrease.

Because Landsat and other source data are sparse prior to 2000, current assessments (including ours) of pre-2000 African wetland conditions remain uncertain, making these discrepancies understandable. Whether African wetland area before 2000 experienced a true declining trend or merely normal interannual fluctuations still requires further study for confirmation.

4. Product evaluation

The wetland maps of this study were also compared with other African wetland maps or datasets. Since the existing wetland thematic maps in Africa were basically single-phase and did not have the results of long-term series, the results that are consistent or close to the previous wetland map production time for verification were only selected, and the results of all 10 periods were not verified. Three wetland thematic datasets, the global regularly flooded wetlands (RFW) [9], the Congo Basin forest wetland dataset (CARPE wetland map) [21] and the World Atlas of Mangroves[11], were used to compare with the results of this study. The wetland classification was verified from the whole and local levels of Africa.

The first was to use RFW to compare and verify all wetland types across Africa. The RFW dataset is a global wetland dataset, which is divided into two wetland

categories: regularly flooding wetlands and lakes. In order to maintain the consistency of the comparison target, all wetland categories in this study were regarded as a wetland category and compared with RFW. The comparison results show that the wetland area (including lakes) of RFW was 1.5325 million square kilometers, while the wetland area (excluding shallow marine waters) in 2019-2021 was 1.1933 million square kilometers, so the area consistency was 77.87 %. From the point of view of the consistency of the distribution of wetlands, 3212 of the 5000 wetland verification points randomly arranged in Africa were consistent with RFW, That is to say, the area of both RFW and this result identified as wetland is about 64.24 % (Supplementary Fig. 17). We could see that the classification map was highly consistent with the RFW dataset in the Congo Basin, some large wetlands in northern Africa and coastal wetlands, and the distribution consistency of some large wetlands can reach more than 80 % (e.g., Banweulu Lake, Supplementary Fig. 18). The inconsistent verification points were mainly concentrated in the periphery of the African Congo Basin and the southern region. We believed that the difference between RFW and this result was mainly that some river-like small wetlands are not extracted by RFW, which may be caused by the insufficient spatial resolution of RFW data. In addition to the overall comparison, the CARPE wetland map and the World Atlas of Mangroves were selected to verify the consistency of the wetlands classification in local areas of Africa. These validations were discussed in detail in the results section of our previous paper "Mapping African wetlands for 2020 using multiple spectral, geo-ecological features and Google Earth Engine" [1], and were not covered here.

Because this study used the TOPMODEL-based diagnostic model to simulate the African inland wetlands in the historical period, the simulation results (with FLDAS soil moisture data as input) could also be used to verify the classification results. On the one hand, the difference between the classification results and the simulation results in the distribution of wetland was compared (Supplementary Fig. 12). By calculating the root mean square error (RMSE) between the wetland area distribution maps based on remote sensing and simulation from 2019 to 2021 (Equations (3)), we found that

there was not much difference between the remote sensing classification and simulation results except in the Congo Basin and several large inland lakes, and the RMSE in about 87 % of the grids was less than 50. On the other hand, the correlation between the simulated wetland time series and the actual classification time series was calculated (Supplementary Fig. 19). The results showed that the Spearman correlation coefficient can reach 0.8500, and the significance is less than 0.01. The high consistency between the remote sensing classification map and the TOPMODEL-based diagnostic model results showed that although the two methods of classification and simulation were different in principle, they both accurately extracted the distribution and change of wetland in Africa, which also proved the accuracy of the classification results from the side.

5. Uncertainty of African wetland classification

For the uncertainty assessment of historical wetland classification in Africa, we referenced the method used by Nicholas J. Murray in Science 'High-resolution mapping of losses and gains of Earth's tidal wetlands', and employed the bootstrapping method to calculate 95% confidence intervals for the classification accuracy and area of wetlands over ten historical periods.

We selected approximately 10% (7,000 sampling points) of the validation points from the accuracy assessment for bootstrapping. Through 1,000 iterations, we calculated the overall accuracy, user accuracy, producer accuracy, omission error, and commission error for wetland classification. The mean of the sampling distribution was reported as the accuracy value, and the 0.025 and 0.975 percentiles of the sampling distribution were used as the 95% confidence interval. This method was implemented using the R package `caret`. Given the asymmetry in omission and commission errors in remote sensing classification, the 95% interval of the resampled omission and commission error distribution was used to estimate the upper and lower bounds of Africa's wetland area. The specific calculation formulas are as follows:

$$area_{lower} = area \times (1 - commission_{P95}) \quad (1)$$

$$area_{upper} = area \times (1 + omission_{P95}) \quad (2)$$

Here, $area$ represents the wetland area value derived from the remote sensing classification map, while $commission_{P95}$ and $omission_{P95}$ are the 95th percentiles of the commission error and omission error in wetland classification, respectively.

6. Effectiveness and uncertainty of inland wetland simulation

The classical TOPMODEL is a semi-distributed rainfall–runoff model based on the Topographic Wetness Index (TWI), originally proposed by Beven and Kirkby (1979). It was developed to simulate soil saturation and surface runoff processes under precipitation recharge. The model uses time series inputs of precipitation (along with other meteorological variables such as evapotranspiration and temperature) and estimates groundwater table depth (WTD) and runoff by calculating infiltration and saturation-excess overland flow, thereby enabling dynamic simulation of wetland extent in response to hydrological changes.

In the TOPMODEL-based diagnostic model employed in Xi et al. (2022) and in this study, precipitation time series is not directly used as the driving input. Instead, multiple reanalysis soil moisture (SM) datasets are used (in place of precipitation), together with soil temperature and freeze–thaw status, to calculate the average water table depth (WTD) for each grid cell. Subsequently, based on the analytical relationship between the Compound Topographic Index (CTI), which reflects topographic control, and the WTD, the inundation threshold CTI* is derived. This allows for the diagnosis of the proportion of inundated/saturated pixels (on a monthly basis) within each grid cell, yielding the wetland fraction f_x, and thus enabling simulation of the spatiotemporal dynamics of wetlands.

In other words, this diagnostic algorithm "decouples" the explicit simulation of precipitation recharge in the original TOPMODEL by implicitly incorporating precipitation forcing into soil moisture reanalysis data, rather than directly treating precipitation as an independent driver. This approach is feasible for two main reasons:

(1) Land Data Assimilation Systems (LDAS) such as FLDAS indirectly account

for the effects of runoff and river inflow on wetland water levels by assimilating remotely sensed soil moisture observations. As a result, the reanalyzed Water Table Depth (WTD) reflects variability driven by non-local precipitation.

(2) Although transient inundation driven by precipitation (e.g., flooded grasslands after heavy rainfall) falls under the broad definition of wetlands, its signal tends to be "averaged out" by short-term fluctuations in multi-decadal time series analysis. The model instead aims to capture long-term saturated zones that are more ecologically important in shaping ecosystem patterns. Thus, using soil moisture reanalysis instead of direct precipitation inputs not only simplifies computation but also emphasizes persistent wetland dynamics.

Two efforts were made to ensure the effectiveness of future inland wetland simulations in this study. One is the effectiveness of the TOPMODEL-based diagnostic model to be verified. As mentioned above, historical wetland remote sensing classification and TOPMODEL simulation were conducted. The results of the two could be applied to verify each other. Based on the TOPMODEL-based diagnostic model and the FLDAS soil moisture data, the wetland time series accurately simulated the area change of wetlands in 2004-2006 and 2016-2018, showing high consistency as a whole (Supplementary Fig. 19). This proved that the TOPMODEL-based diagnostic model used in this study can accurately simulate the area change of wetland when the input soil moisture data was accurate. The other is the validity of CMIP6 input climate data to be verified. The CMIP6 data was based on data produced by climate models. In this study, SM data from 14 models were used and showed great differences. To ensure the validity of the simulation in the future period, the SM mean time series of 14 models under the 'historical' scenario were compared with the SM time series based on FLDAS (Supplementary Fig. 20). We found that with FLDAS data as the real value reference, although the SM mean did not show corresponding changes in a specific year (the middle year 1998, 2005, and 2011) and the sensitivity is not high, it can unexpectedly show a good consistency with FLDAS data in the general trend. That is to say, although the simulation results with 14 soil moistures of CMIP6 are very different and could not

accurately simulate the change of wetland area in a specific drought or flood year, its mean value could predict the long-term trend of wetland area in Africa in the future, which was also meaningful.

There were also uncertainties in this future inland wetland simulation. First, the TOPMODEL-based diagnostic model itself has some defects. The TOPMODEL-based diagnostic model is based on the CTI threshold to divide the wetland range. CTI reflects the spatial distribution of soil moisture and surface saturation and the process of runoff generation which is the core component of TOPMODEL. However, simulations with CTI would get obviously unreasonable results when applied to flat terrain areas. For example, the CTI of the valley area is only higher on the narrow catchment line, so the simulated wetland range is mostly linear, which results in that the model cannot simulate some lake wetlands and valley wetlands well. In addition, due to the limitation of experimental efficiency and computer performance, the value of M in the model was limited to an integer of 1-15. The actual situation may require M to further expand the scope and have a more accurate decimal place, which also reflects the fact that the previous simulation cannot simulate the actual wetland perfectly. Second, the input climate data and the corrected wetland map itself also had certain errors. As mentioned above, due to the problem of the accuracy of the existing forecast climate data, this simulation could only simulate the general trend of future changes in Africa's wetlands, but could not predict changes in wetland area caused by drought or floods (extreme climate) in a specific year. Moreover, due to the serious shortage and inaccuracy of the current number of wetland thematic maps in Africa, in addition to the wetland maps produced in this study, the correction of model parameters by relying on wetland maps with relatively large errors (such as RFW or other simulated wetland datasets) could not guarantee the accuracy of simulation. Third, the direct impact of human activities on wetlands could not be well predicted. This study only predicted the future changes of inland wetlands in Africa based on CMIP6 climate data, which is based on the conclusion that the changes of inland wetlands in Africa during 1984-2021were closely related to climate factors. However, we found that the human footprint of Africa,

whether inland or coastal areas, showed a growth trend after 2000. This showed that the future inland wetlands would be direct affected by human activities (infrastructure development, land conversion, and water withdrawal), and the impact would increase year by year. Therefore, there was still considerable uncertainty in the future changes of wetlands, which are caused by the combined effects of climate and human activities.

Additionally, during the historical period, the TOPMODEL-based diagnostic framework benefited from the FLDAS soil moisture fields, which implicitly incorporated signals of river inflow and floodplain inundation through data assimilation. However, this assimilation capability is unavailable in the climate change (future) simulations. The diagnostic model is driven solely by meteorological forcing (soil moisture) derived from global climate models (GCMs), and thus mainly reflects local soil water balance and topographic control. Consequently, fluvially-driven hydrological processes—particularly river routing and upstream runoff contributions—are not explicitly represented in these future simulations. This limitation may lead to an underestimation of variability in river-fed wetlands such as the Sudd and the Niger Inland Delta. Although the TOPMODEL-based diagnostic approach effectively captures long-term wetland dynamics governed by local soil moisture and terrain, it does not explicitly account for river-driven inundation. We recognize this as an important source of uncertainty and emphasize that future studies should couple the diagnostic framework with dynamic river routing or surface water models to better represent fluvial processes under changing climate conditions.

Furthermore, we acknowledge that while the TOPMODEL-based diagnostic model efficiently utilizes the compound topographic index (CTI) and reanalyzed soil moisture (SM) to estimate long-term saturation and inundation fractions at the grid scale, it does omit explicit simulations of river routing and floodplain processes, nor does it directly incorporate precipitation time series as input. Additionally, the model cannot resolve microtopography and small-scale wetland dynamics, nor does it account for anthropogenic regulation (e.g., dams, irrigation). Consequently, it exhibits notable limitations in simulating floodplains primarily driven by riverine inputs or extreme

precipitation (e.g., the Sudd Wetland). Future improvements should involve coupling with river routing models, employing higher-resolution DEMs, and integrating direct precipitation forcing.

7. Wetland sampling and classification feature construction

The purpose of sampling was to generate training datasets and validation datasets for wetland classification. The current study's sampling method adopted the traditional remote sensing visual interpretation method, and the researchers judged the wetland categories of the sampling point based on the multi-source remote sensing image. Before sampling, we began the study's data collection process by downloading Landsat remote sensing images of 10 periods based on the GEE platform, as well as Digital Elevation Model (DEM) and vegetation index images for auxiliary discrimination. The sampling points were uniformly arranged following the method of systematic sampling. Covering the entire continent of Africa, a grid containing 270,000 blank vector points at an interval of 0.1° (latitude) × 0.1° (longitude) was generated (Supplementary Fig. 2). The initial attribute of each vector point was defaulted to highland. The researchers referred to the Landsat, DEM, and vegetation water index images and visually discriminated each vector point with the help of Google Earth's high-resolution images. If the sampling point fell within the wetland area, the wetland categories were judged according to the African Wetland Classification System, and its attributes were modified. If it was not located within the wetland area, the default state was left unchanged. In some cases, we moved the position of the sampling point slightly so that the sampling point position could represent wetland better.

Constructing accurate, comprehensive wetland remote sensing features can effectively improve the accuracy of classification. This study followed the method proposed by Murray et al. (2019)[14] in calculating the classification feature set, including the Landsat feature band, vegetation index, terrain feature, and hydrological feature. For the Landsat feature band, the six bands of red, green, blue, near-infrared, short-wave infrared 1, and short-wave infrared 2 were selected as input[2]. Based on the

consideration that the wetland ecosystem has two different characteristics of vegetation and hydrology, the vegetation index and water index were calculated as supplements. The vegetation index comprised a normalized vegetation index (NDVI)[33] and enhanced vegetation index (EVI)[34]. The water index included a normalized water index (NDWI)[35] and improved normalized water index (MNDWI)[36]. Because the single-temporal remote sensing images were not enough to distinguish wetlands from other land cover categories and could not distinguish different categories of wetlands[37], the spectral-temporal features of wetlands were extracted. All Landsat 4/5/7/8 images from 1984 to 2021 across Africa were collected and summarized into 10 periods of cloudless spatial-temporal composite measurement layers. Based on these cloud-free temporal composite metric layers, the maximum, minimum, standard deviation, median, percentiles at 10%, 25%, 50%, 75%, and 90%, and the mean between 0-10%, 10-25%, 25-50%, 50-75%, 75-90%, and 90-100% of the six Landsat feature bands and four spectral indices were calculated in 10 periods as the spectral-temporal characteristics of wetlands. Due to the insufficient number of early Landsat images, in order to enable remote sensing images to cover all of Africa, we defined the earliest three periods as longer in the allocation of the 10 periods, which were 7-year and 5-year. After 2000, the images were synthesized according to a time span of 3-year (Supplementary Table 5). To improve the classification accuracy and further distinguish wetlands from highlands, we added topographic and hydrological variables, such as DEM, slope, mountain shadow, and flow distance, to the classification features[38]. The flow distance was defined as the horizontal or vertical component of the downhill distance of each pixel along the flow path to the pixel on the river they flow into based on DEM[21,39].

8. Soil moisture and other wetland simulation data

The observed soil moisture data were drawn from the Famine Early Warning Systems Network Land Data Assimilation System (FLDAS) dataset, which was designed to facilitate food security assessment in developing countries with limited data[40]. It includes information on many climate-related variables, including water content,

humidity, evaporation, average soil temperature, and total precipitation rate. The SM data resolution in FLDAS is 11,132 m, divided into four vertical soil layers (0-0.1, 0.1-0.4, 0.4-1, and 1-2 m).

CMIP6 simulated climate data were also used in this study. CMIP6 contains the results of 122 different climate models from 45 different institutions. Soil moisture data under "historical" (1850-2014) and four SSP future scenarios (2015-2100) in 14 models of the CMIP6 database were downloaded for wetland simulation. In particular, the simulation of historical scenarios is a historical climate simulation beginning with 1850 driven by observation-based, time-varying external forcing, including various natural and anthropogenic climate forces[41]. Four SSPs scenarios were adopted from the first layer of ScenarioMIP: SSP1-2.6 (low forcing sustainability pathway), SSP2-4.5 (medium forcing middle-of-the-road pathway), SSP3-7.0 (medium- to high-end forcing pathway), and SSP5-8.5 (high-end forcing pathway). The SM data of the above 14 models in the "historical" and 4 SSP future scenarios together constituted a CMIP6 multi-model ensemble in the current study.

Before the simulation, the relevant data were downloaded, including African watershed vector data, CTI data, and soil moisture data. Note that this simulation is not based on the latitude and longitude grid, but uses the watershed as the basic unit for simulation. The Africa watershed data was derived from the WWF HydroSHEDS Basins Level 8 dataset[42], which ranges from Level 1 (coarse) to Level 12 (detailed). The 8-level watershed data used in this study have a total of 41745 watershed units in Africa. The CTI data was derived from the results of Marthews *et al.* [43], with a spatial resolution of 500 m, excluding most river, lake and reservoir areas.

9. TOPMODEL-based diagnostic model

Based on the formation mechanism of wetlands or the interaction mechanism between wetlands and their surrounding surface types, the wetland model realized the simulation and prediction of wetland area and spatial distribution, which provided a new direction for obtaining global wetland information. Among many wetland models, the

TOPMODEL method is the most recognized and commonly used wetland model because its parameters have clear physical meaning and focus on the formation mechanism of wetlands[9,44,45].

In this study, the TOPMODEL-based diagnostic model was used to predict future wetland changes. The main simulation process referred to the methods introduced in the paper of Yi Xi *et al.* (2020). The model has successfully predicted the spatial distribution, range, and seasonal variation of natural wetlands. According to the principle of TOPMODEL method, assuming that the local hydraulic gradient is approximated by the local topographic slope and the water-table variations can be assimilated to a succession of steady states with uniform recharge, the analytical relationship between the water table and the local topographic index distribution of the watershed can be expressed by the following formula:

$$CTI_i - \overline{CTI_x} = -M(\Gamma_i - \overline{\Gamma_x}) \tag{3}$$

CTI is the composite terrain index, which is defined as the logarithm of the ratio of contribution area to local slope[43]. x and i represent the watersheds and pixels numbering respectively. CTI_i represents the value of the composite topographic index of the pixel i, and $\overline{\mathrm{CTI}_x}$ represents the average CTI of the watershed x. Γ_i represents the water table depth (WTD) of the pixel i, and $\overline{\Gamma_x}$ represents the average water table depth of the watershed x. In view of the different internal characteristics of topography and soil types in different watersheds, M is an adjustable parameter describing the decrease of soil transmissivity with depth. The M values of different watersheds were different, and the value range is set to 1-15. When the groundwater level of pixel i is located on the soil surface (Γ_i = 0), the CTI threshold of a pixel in the watershed x is derived:

$$CTI_x^* = \overline{CTI_x} + Ml \times \overline{\Gamma_x} \tag{4}$$

Pixels with CTI_i greater than the threshold CTI_x^* are considered to be the submerged part of the watershed x, and the wetland area is the accumulation of all the submerged part pixel areas. The M value should be determined based on the actual wetland area. The specific method is to calculate the RMSE of the wetland area

(observed value) calculated by the existing wetland map and the simulated wetland area (simulated value) in the historical period, and select the M value used in the simulation when the RMSE is the smallest as the real M value of the watershed for subsequent wetland simulation in the future. The RMSE calculation formula is as follows:

$$RMSE = \sqrt{\frac{\sum_{i=1}^{n}(O_i - P_i)^2}{n}} \quad (5)$$

Where n is the number of wetland maps in different periods, O_i and P_i are the observed wetland area and the simulated wetland area in the corresponding period, respectively. The calculation of WTD in the study was also based on the definition given in the paper of Yi Xi et al. (2020). WTD was defined as an index composed of saturated volumetric water content and 'actual' soil depth masked by soil freezing / thawing state and soil temperature.

$$\Gamma_x = Z_{l_0} - \sum_{l=1}^{l_0} \theta_l \cdot \frac{\Delta z_l}{\theta_s} \quad (6)$$

The subscript l represents the lth soil layer, l_0 is the number of layers from the top (surface, l = 1), θ_l is the soil moisture (SM) of the layer l, Δz_l is the thickness of the layer l, and θ_s is the saturated volumetric water content (uniform over depth).

10. Analysis of the Correlation Between Multi-Source Precipitation Data and Wetland Extent at the Subcontinental Scale

Interannual variability of African precipitation exhibits a high degree of uncertainty across different datasets, and spatially the changes in African rainfall from 1980 to 2020 do not display continent-wide homogeneity. Therefore, we have supplemented our study with a Spearman correlation analysis between precipitation and wetland area at the subcontinental scale.

We employed four precipitation datasets: (1) ERA5-Land; (2) GPCP (Global Precipitation Climatology Project); (3) TerraClimate; and (4) FLDAS. Seven representative subcontinental wetlands in Africa were selected: (1) the Congo Basin; (2) the Inner Niger Delta; (3) the Lake Chad region; (4) the Sudd Wetland and surrounding areas; (5) the Zambezi River Basin; (6) Lake Bangweulu; and (7) the Okavango Delta.

Their precise extents are illustrated in the Supplementary Figure. 21.

We assessed the significance of the interannual trends in both wetland area (with missing values linearly interpolated) and precipitation (using the mean of the four datasets) over the 1980–2020 period (Supplementary Table 6). At the subcontinental scale, most of these characteristic wetland regions in Africa displayed significant monotonic trends in precipitation. In particular, the Congo Basin in central Africa exhibited a significant decreasing trend, whereas the Inner Niger Delta in the Sahel region showed a significant increasing trend. Correspondingly, the wetland areas in these regions—such as the forest wetlands of the Congo Basin and the herbaceous marshes of the Niger River—demonstrated significant declines and increases, respectively.

To quantitatively evaluate the relationship between wetland area and monthly cumulative precipitation (mm), we computed Spearman's rank correlation coefficients (ρ) and associated p-values. Among the seven selected subcontinental wetlands, five exhibited statistically significant correlations of moderate strength ($0.3 \leq |\rho| < 0.7$) between area and precipitation. The Okavango Delta and Lake Bangweulu wetlands did not show significant correlations with precipitation changes. Moreover, none of the seven regions demonstrated a strong correlation ($|\rho| \geq 0.7$) between wetland area variation and precipitation.

ERA5-Land is available from https://developers.google.com/earth-engine/datasets/catalog/ECMWF_ERA5_LAND_MONTHLY_AGGR. GPCP is available from https://www.ncei.noaa.gov/products/global-precipitation-climatology-project. TerraClimate is available from https://developers.google.com/earth-engine/datasets/catalog/IDAHO_EPSCOR_TERRACLIMATE. FLDAS is available from https://developers.google.com/earth-engine/datasets/catalog/NASA_FLDAS_NOAH01_C_GL_M_V001.

Supplementary Tables

Supplementary Table 1 | African wetland classification system for remote sensing.

Category I	Category II	Description
Inland wetland	Inland marsh	Natural wetland with dominant herbaceous vegetation in inland areas
	Inland swamp	Natural wetland with dominant woody vegetation in inland areas including forested wetland and shrub wetland
	Inland salt pan	Natural wetlands without vegetation cover
	Inland surface water	Natural open waterbody in inland areas including lake and river
Coastal wetland	Coastal marsh	Natural wetland with dominant herbaceous vegetation in coastal areas
	Coastal swamp	Natural wetland with dominant woody vegetation in coastal areas including forested wetland and shrub wetland
	Tidal flat	The intertidal flat with no or very low vegetation coverage including sand beach, rocky shore, and coral reef
	Shallow marine water	Marine waterbody between coastline and the contour of 6-m depth

Supplementary Table 2 | A list of CMIP6 models for future inland wetland prediction in this study. Only those CMIP6 models that can provide soil moisture data under historical and SSP scenarios (SSP126, SSP245, SSP370, and SSP585) were selected for research.

Model	Historical	SSP126	SSP245	SSP370	SSP585
ACCESS-CM2	√	√	√	√	√
ACCESS-ESM1-5	√	√	√	√	√
BCC-CSM2-MR	√	√	√	√	√
CAS-ESM2-0	√	√	√	√	√
CESM2-WACCM	√	√	√	√	√
CMCC-CM2-SR5	√	√	√	√	√
CMCC-ESM2	√	√	√	√	√
CanESM5	√	√	√	√	√
FGOALS-f3-L	√	√	√	√	√
MIROC6	√	√	√	√	√
MRI-ESM2-0	√	√	√	√	√
NorESM2-LM	√	√	√	√	√
NorESM2-MM	√	√	√	√	√
TaiESM1	√	√	√	√	√

Supplementary Table 3 | The statistics of wetland areas in Africa in the historical period, including the lost area, increased area, net change, area change rate over 38 years.

Class	Area in 1984 (10,000 km^2)	Area in 2021 (10,000 km^2)	Lost area	Increased area	Net change in area	area change rate
Surface water	28.97	30.07	1.71	2.82	1.11	3.82%
Inland Marsh	34.29	34.22	6.81	6.74	-0.08	-0.22%
Inland Swamp	41.81	41.15	5.16	4.49	-0.67	-1.60%
Inland Salt pan	3.05	3.23	0.34	0.52	0.18	5.80%
Coastal swamp	4.63	4.19	0.77	0.33	-0.44	-9.50%
Tidal flat	2.23	1.96	0.58	0.30	-0.27	-12.20%
Coastal Marsh	5.09	4.65	1.16	0.72	-0.44	-8.65%
Shallow marine water	13.05	14.65	0.18	1.78	1.60	12.28%
Inland wetlands	108.13	108.67	12.20	12.74	0.54	0.50%
Coastal wetlands	11.95	10.80	2.08	0.93	-1.15	-9.64%
Africa wetlands	120.08	119.47	13.85	13.24	-0.61	-0.51%

Supplementary Table 4 | The remote sensing error matrix for the two periods of 1984-1990 and 2019-2021.

(1) Error matrix of the overall wetland from 1984 to 1990

Class	Highlands	Wetlands	Total	User's accuracy	Kappa
Highlands	59619	2143	61762	96.53%	
Wetlands	703	7583	8286	91.52%	
Total	60322	9726	70048		
Producer's accuracy	98.83%	77.97%		95.94%	
Kappa					0.955

(2) Error matrix of the overall wetland from 2019 to 2021

Class	Highlands	Wetlands	Total	User's accuracy	Kappa
Highlands	63607	1490	65097	97.71%	
Wetlands	606	8220	8826	93.13%	
Total	64213	9710	73923		
Producer's accuracy	99.06%	84.65%		97.16%	
Kappa					0.963

(3) Error matrix for 8 wetland categories from 1984 to 1990

Class	Highlands	Surface water	Marsh	Inland swamp	Inland Salt pan	Coastal swamp	Tidal flat	Total	User's accuracy
Highlands	59619	454	1081	461	30	34	83	61762	96.53%
Surface water	120	2525	93	15	9	12	36	2810	89.86%
Marsh	340	96	2158	45	0	12	4	2655	81.28%
Inland swamp	185	10	39	1459	0	9	0	1702	85.72%
Inland Salt pan	5	0	0	0	151	0	0	156	96.79%
Coastal swamp	26	5	6	8	0	376	2	423	88.89%
Tidal flat	27	11	2	0	3	6	491	540	90.93%
Total	60322	3101	3379	1988	193	449	616	70048	0
Producer's accuracy	98.83%	81.43%	63.87%	73.39%	78.24%	83.74%	79.71%	0	95.33%
Kappa	0	0	0	0	0	0	0	0	0.86

(4) Error matrix for 8 wetland categories from 2019 to 2021

Class	Highlands	Surface water	Marsh	Inland swamp	Inland Salt pan	Coastal swamp	Tidal flat	Total	User's accuracy
Highlands	63607	115	763	460	36	21	95	65097	97.71%
Surface water	76	2991	45	3	4	3	26	3148	95.01%
Marsh	304	37	2474	57	0	9	3	2884	85.78%
Inland swamp	182	8	18	1489	0	16	0	1713	86.92%
Inland Salt pan	8	2	0	0	141	0	1	152	92.76%
Coastal swamp	13	3	3	8	0	381	0	408	93.38%
Tidal flat	23	12	0	0	6	1	479	521	91.94%
Total	64213	3168	3303	2017	187	431	604	73923	0
Producer's accuracy	99.06%	94.41%	74.90%	73.82%	75.40%	88.40%	79.30%	0	96.81%
Kappa	0	0	0	0	0	0	0	0	0.86

Supplementary Table 5 | The time span of wetlands remote sensing classification of 10 periods and wetland simulation of the 17 periods from 1984 to 2100.

Results	Number	Middle year	Start time	Ending time
Classification	1	1987	1984.01.01	1990.12.31
	2	1993	1991.01.01	1995.12.31
	3	1998	1996.01.01	2000.12.31
	4	2002	2001.01.01	2003.12.31
	5	2005	2004.01.01	2006.12.31
	6	2008	2007.01.01	2009.12.31
	7	2011	2010.01.01	2012.12.31
	8	2014	2013.01.01	2015.12.31
	9	2017	2016.01.01	2018.12.31
	10	2020	2019.01.01	2021.12.31
Simulation	1	2018	2015.01.01	2020.01.01
	2	2023	2021.01.01	2025.01.01
	3	2028	2026.01.01	2030.01.01
	4	2033	2031.01.01	2035.01.01
	5	2038	2036.01.01	2040.01.01
	6	2043	2041.01.01	2045.01.01
	7	2048	2046.01.01	2050.01.01
	8	2053	2051.01.01	2055.01.01
	9	2058	2056.01.01	2060.01.01
	10	2063	2061.01.01	2065.01.01
	11	2068	2066.01.01	2070.01.01
	12	2073	2071.01.01	2075.01.01
	13	2078	2076.01.01	2080.01.01
	14	2083	2081.01.01	2085.01.01
	15	2088	2086.01.01	2090.01.01
	16	2093	2091.01.01	2095.01.01
	17	2098	2096.01.01	2100.01.01

Supplementary Table 6 | Correlation Between Precipitation and Wetlands in Africa. Trend significance was assessed with the Mann–Kendall nonparametric test; the table reports the *S* statistic, the standardized *Z* and two-sided *P*-values, and Sen's slope as the robust estimate of trend magnitude (units per year). Spearman's rank correlation (ρ) was used to evaluate the association between regional precipitation and wetland area, with two-sided *P*-values reported.

（1）Trend analysis of wetland area changes

Region	S-statistic	Z-score	p-value	Sen's Slope	Significance
1	-542	-6.076479	1.23E-09	-663.4078	Significant
2	688	7.716341	1.2E-14	107.6894	Significant
3	566	6.346045	2.21E-10	167.5459	Significant
4	-584	-6.54822	5.82E-11	-270.0125	Significant
5	-80	-0.887323	0.374905	-3.077	Not significant
6	336	3.762699	0.000168	19.80628	Significant
7	-164	-1.830806	0.06713	-16.11553	Not significant

（2）Trend analysis of precipitation changes

Region	S-statistic	Z-score	p-value	Sen's Slope	Significance
1	-488	-5.469953865	4.5E-08	-0.613137	Significant
2	300	3.358349499	0.000784	0.229096	Significant
3	256	2.864144221	0.004181	0.245298	Significant
4	-248	-2.774288716	0.005532	-0.240429	Significant
5	202	2.257619563	0.023969	0.377895	Significant
6	130	1.448920018	0.14736	0.167962	Not significant
7	160	1.785878161	0.074119	0.259604	Not significant

（3）Spearman's rank correlation tests

Region	Wetland Area (km^2)	Precipitation (mm)	Spearman's ρ	p-value	Significance
1	296311.3479	145.1321746	0.680139373	2.15E-06	Significant
2	7708.180048	36.42429107	0.555052265	0.000211	Significant

3	15492.05352	38.61809293	0.425958188	0.005866	Significant
4	86741.74304	79.58088573	0.342160279	0.02909	Significant
5	5117.687515	75.65805395	0.330313589	0.035449	Significant
6	12513.2459	99.07811464	0.294076655	0.062359	Not significant
7	5318.688789	42.87566381	0.113240418	0.4795	Not significant

*Wetland area and precipitation values represent means across nine temporal periods

Supplementary Figures

Supplementary Figure. 1 | Area of African wetland study. a, African wetland classification area. The Yellow area is the main distribution area of wetlands in Africa, which accounts for more than 95 % of the total wetland area. b, the specific boundary between inland wetlands and coastal wetlands.

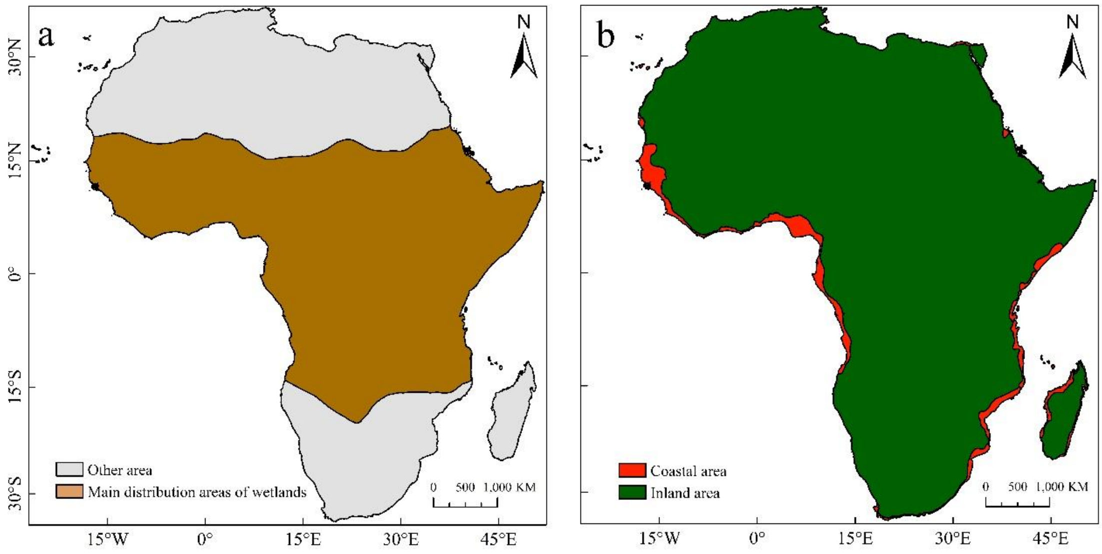

Supplementary Figure. 2 | Distribution map of wetlands sampling points in Africa. Highland's sampling points are not shown in the figure.

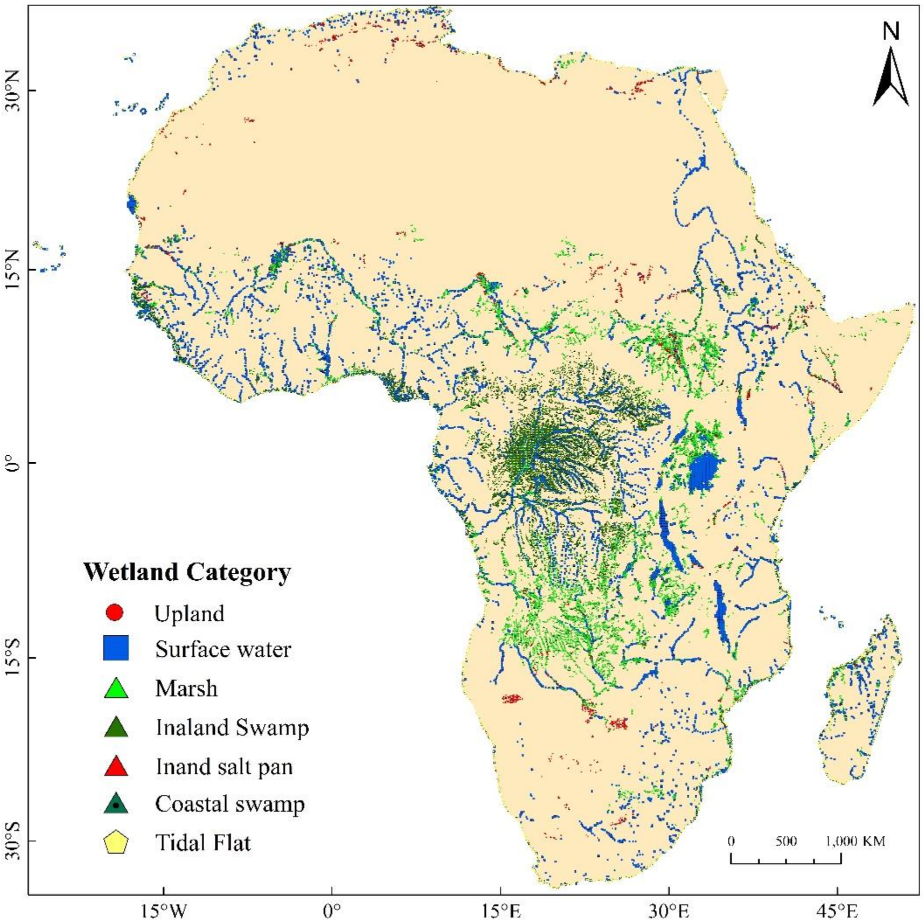

Supplementary Figure. 3 | Diagram of workflow for simulating future changes of African inland wetlands. It mainly includes three parts: classification of historical period, simulation of historical period, and simulation of future period.

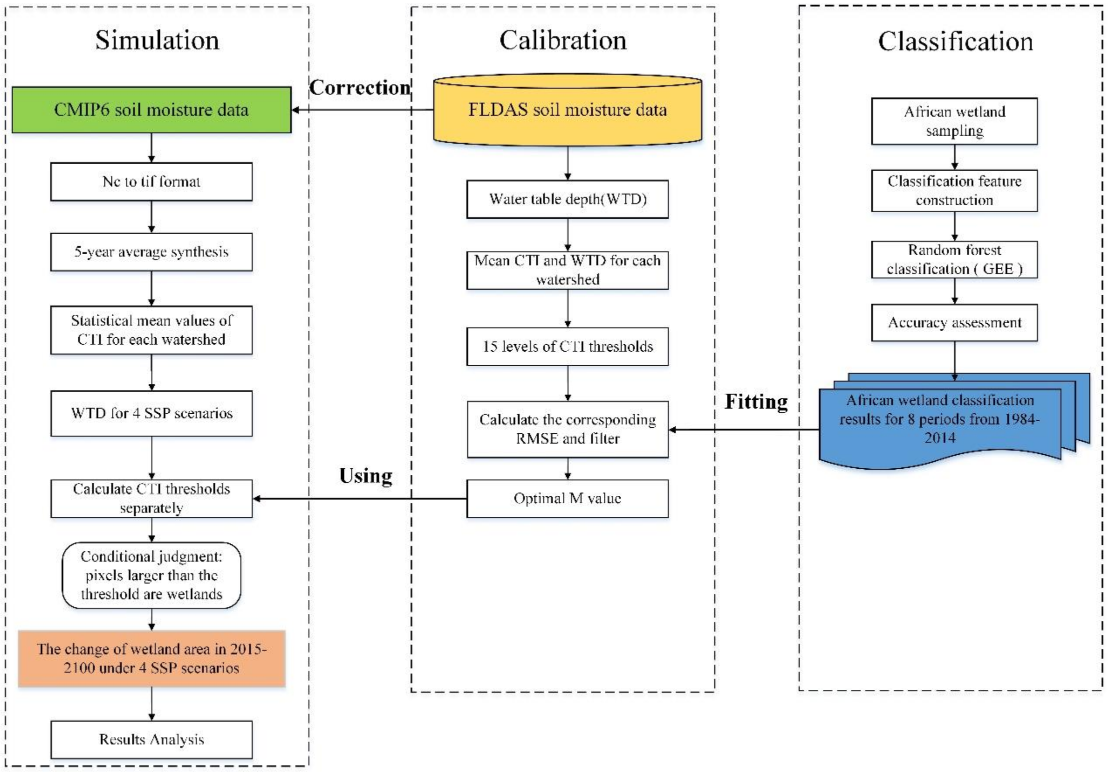

Supplementary Figure. 4 | The gain, loss and net change area of wetland in Africa during 1984-2021. Net change area is the area of wetland gains minus losses. Area statistics were calculated using 0.2° grid cells.

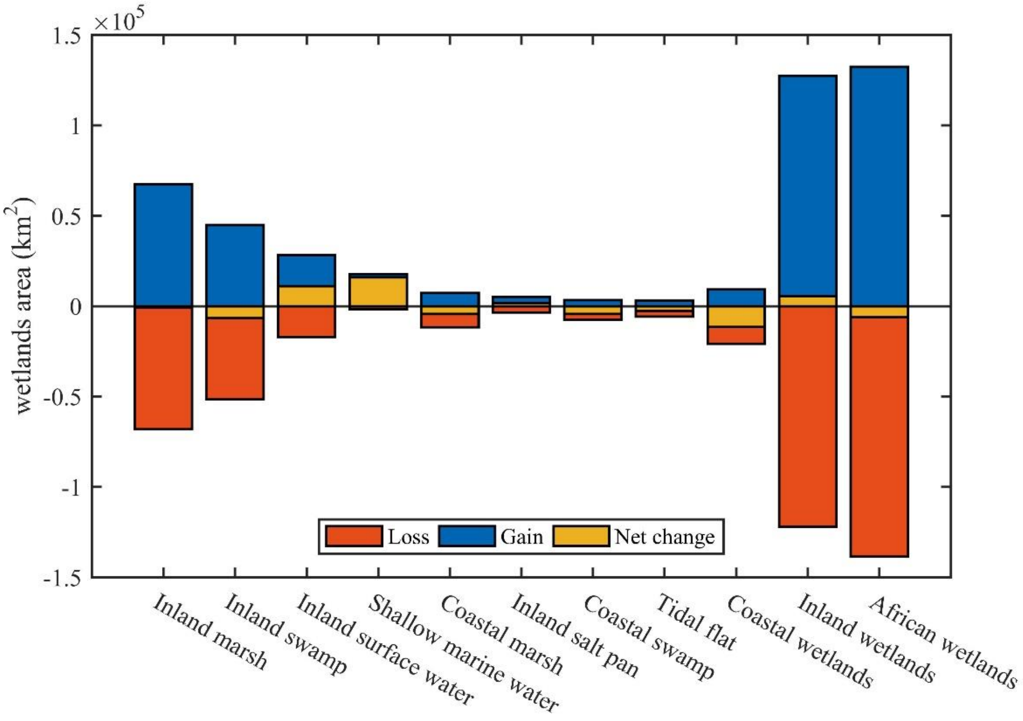

Supplementary Figure. 5 | Wetland loss map by country from 1984 to 2021. National border data was derived from large scale international boundary (LSIB) data.

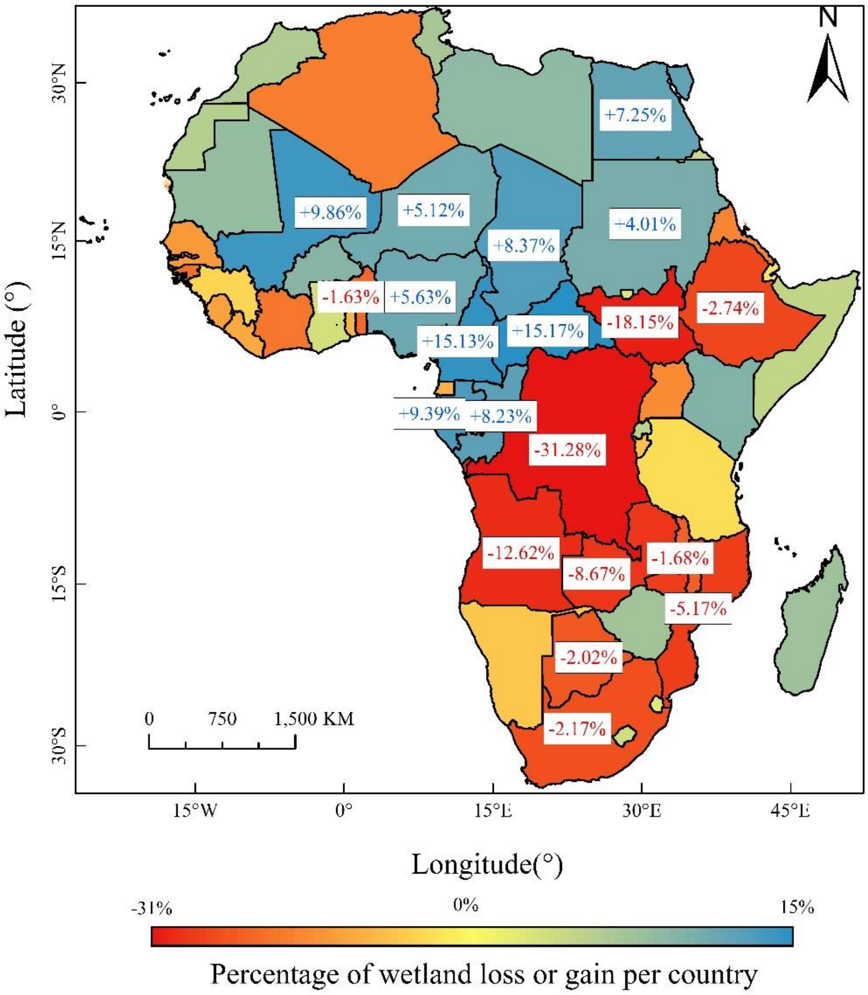

Supplementary Figure. 6 | Time Series and trends of area changes in African wetlands from 1984 to 2021. a-c show the time series and trends for all African wetlands, inland wetlands only, and coastal wetlands only. The linear trend was calculated since 2000. P-value and Z value (two-sided) were obtained by the Mann-Kendall method.

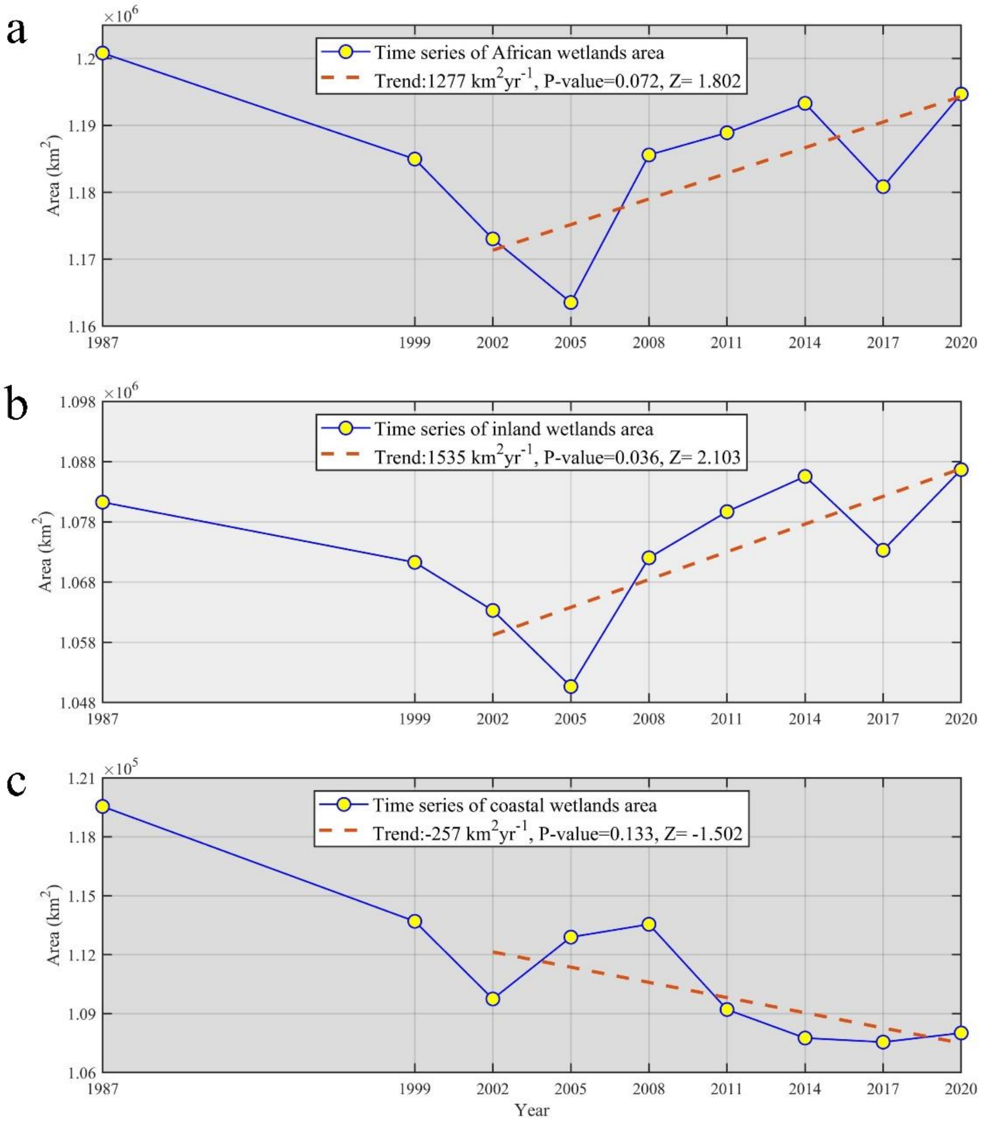

Supplementary Figure. 7 | Global Human Footprint map in 2020. The spatial resolution is 1 km and pixel values represent the total score of human-activity impact. The dataset originates from Mu et al. (2022)[46] and is published under the Creative Commons Attribution 4.0 International (CC BY 4.0) license; see https://creativecommons.org/licenses/by/4.0/.

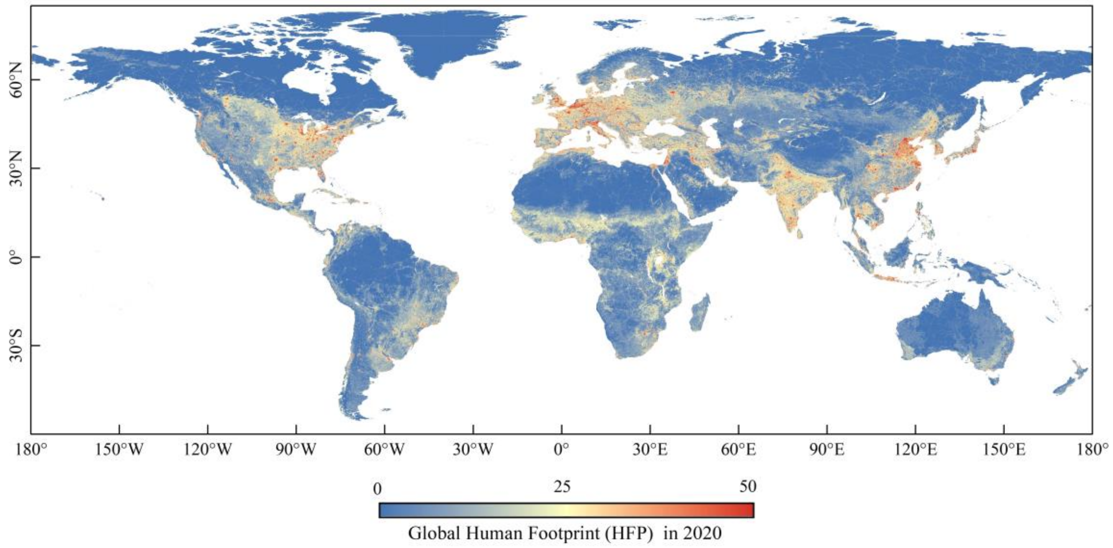

Supplementary Figure. 8 | Comparison of HFP between coastal wetlands area and inland wetlands area in Africa after 2000. HFP was averaged over the same time span as the classification. The horizontal coordinates are the middle years of the seven periods after 2000, and the vertical coordinates are the HFP values. P-value (two-sided) was obtained by the Mann-Kendall method. The HFP dataset originates from Mu et al. (2022)[46] and is published under the Creative Commons Attribution 4.0 International (CC BY 4.0) license; see https://creativecommons.org/licenses/by/4.0/.

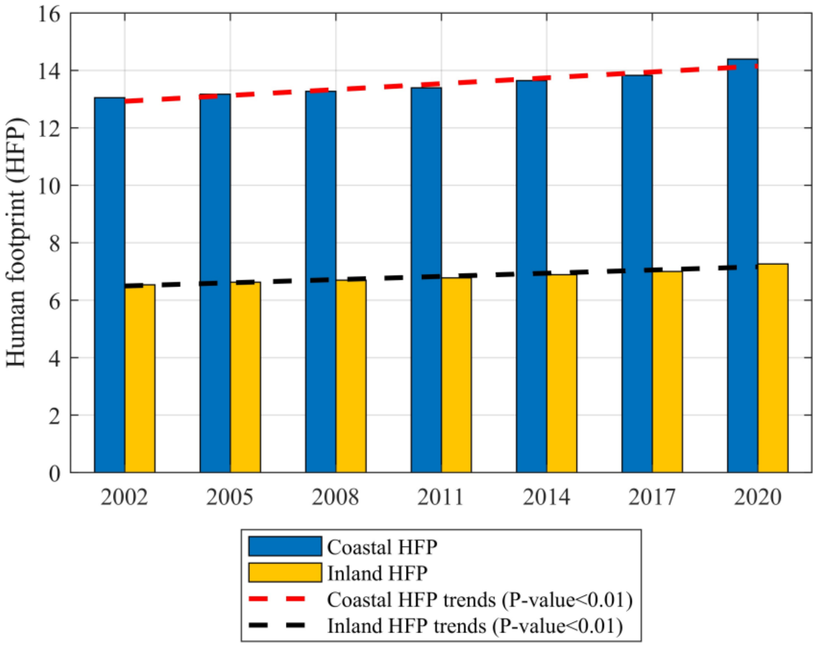

Supplementary Figure. 9 | The time series comparison of inland swamp area and drought index PDSI in the Congo Basin. The time span is 1984–2021. Time points correspond to the midpoints of nine historical periods.

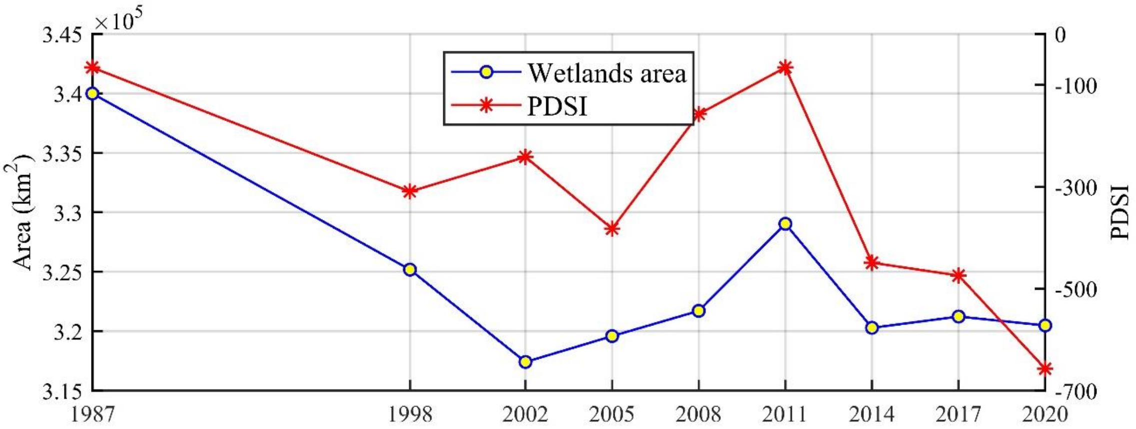

Supplementary Figure. 10 | A box plot of area changes for inland wetlands in Africa based on soil moisture data of 14 models in the historical and future periods. The time span of wetland area change in historical period is from 1984 to 2014, and the time span of four SSP scenarios is from 2015 to 2100.

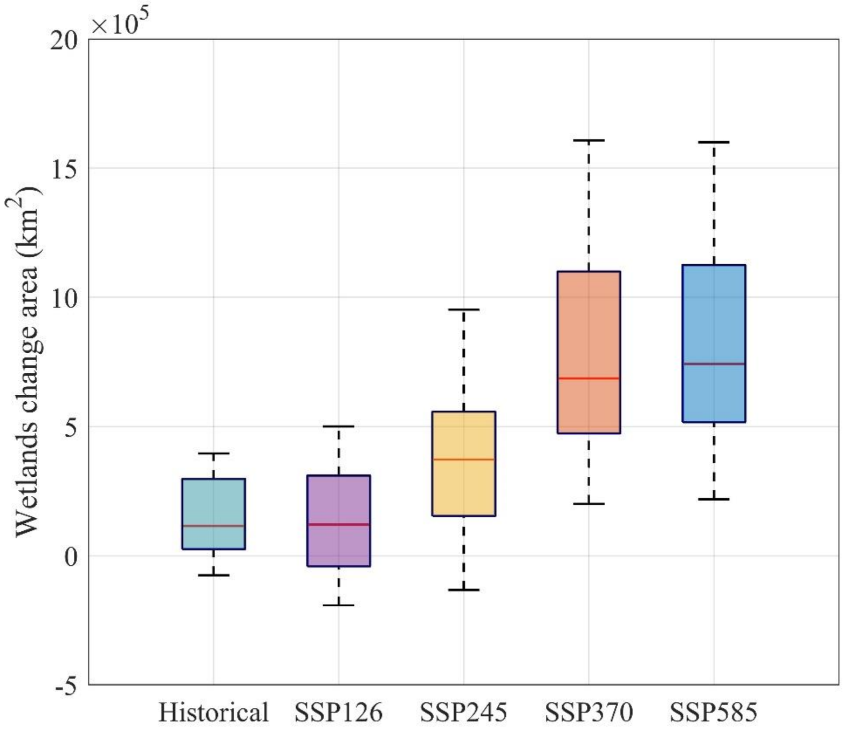

Supplementary Figure. 11 | The gain, loss and net change of inland wetland area in Africa under four SSP scenarios from 2015 to 2100. Net change area is the area of wetland gains minus losses. Area statistics were calculated using 0.2° grid cells.

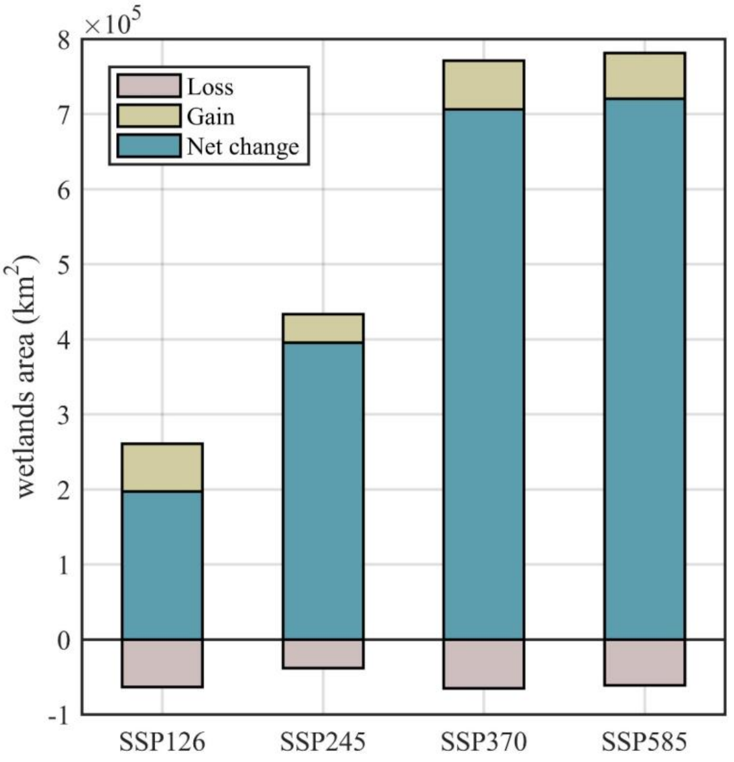

Supplementary Figure. 12 | Spatial distribution map of area relative error between simulated wetland and classified wetland in historical period. a, The RMSE between the simulation results based on FLDAS soil moisture data and the remote sensing classification results. b, The RMSE between the simulation results based on CMIP 6 soil moisture data and the remote sensing classification results.

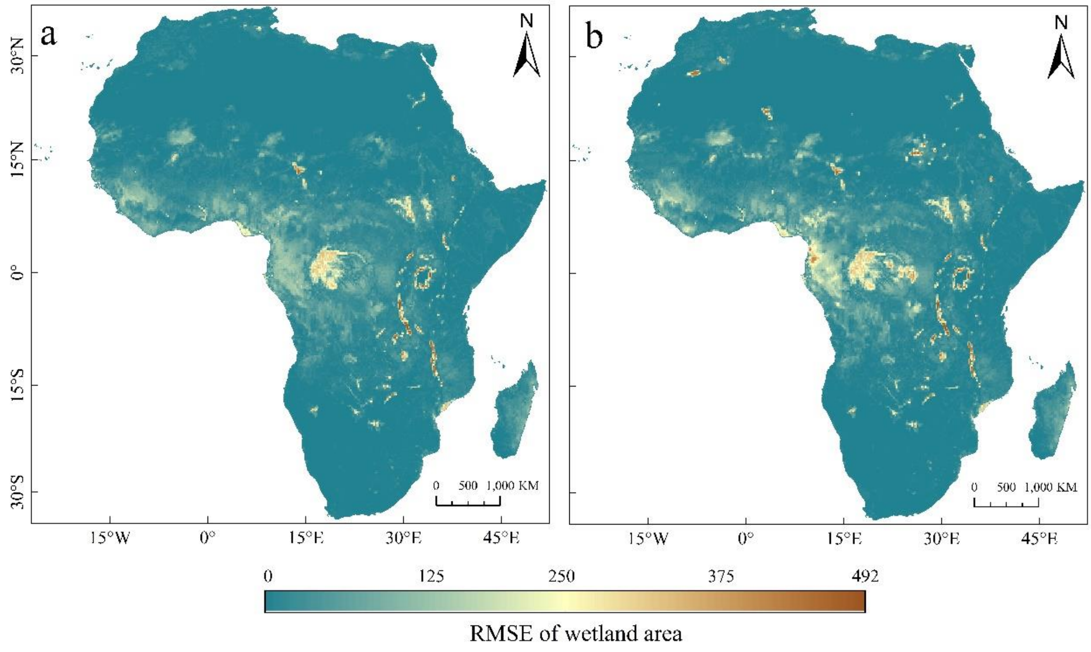

Supplementary Figure. 13 | Accuracy of African wetland remote sensing classification in 10 periods (1984-2021). From 1987 to 2020, classification accuracy increased with the number and quality of imagery.

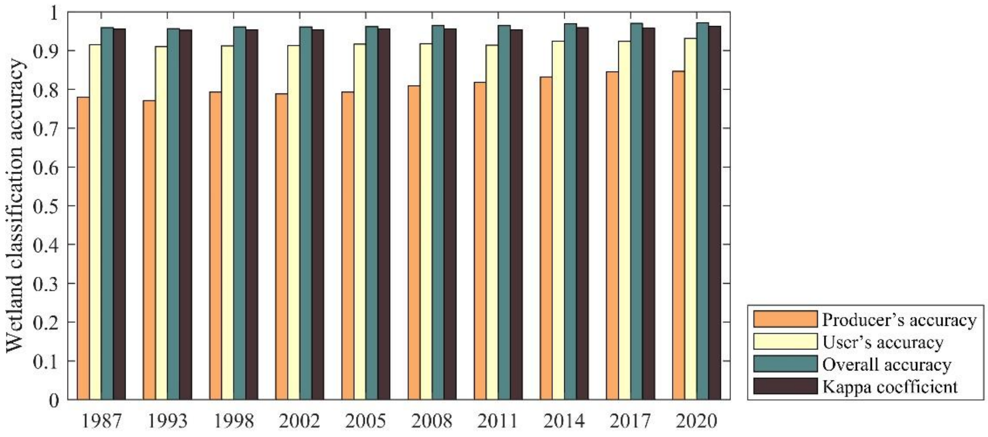

Supplementary Figure. 14 | The spatial distribution of temperature and precipitation changes under the SSP126 scenario. The time span covers the period from 2015 to 2100.

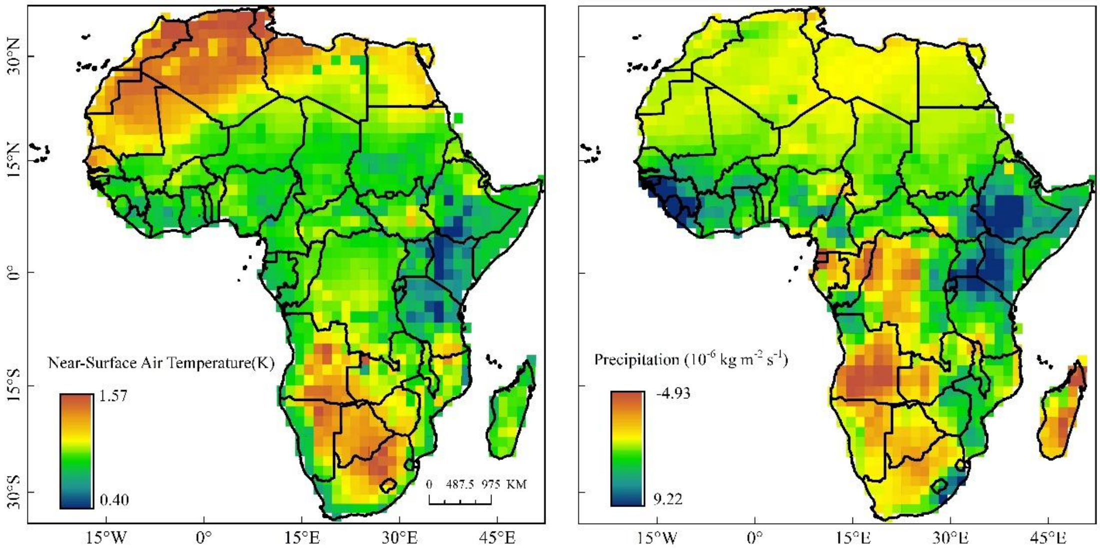

Supplementary Figure. 15 | The spatial distribution of soil moisture changes under the SSP126 scenario. The time span covers the period from 2015 to 2100.

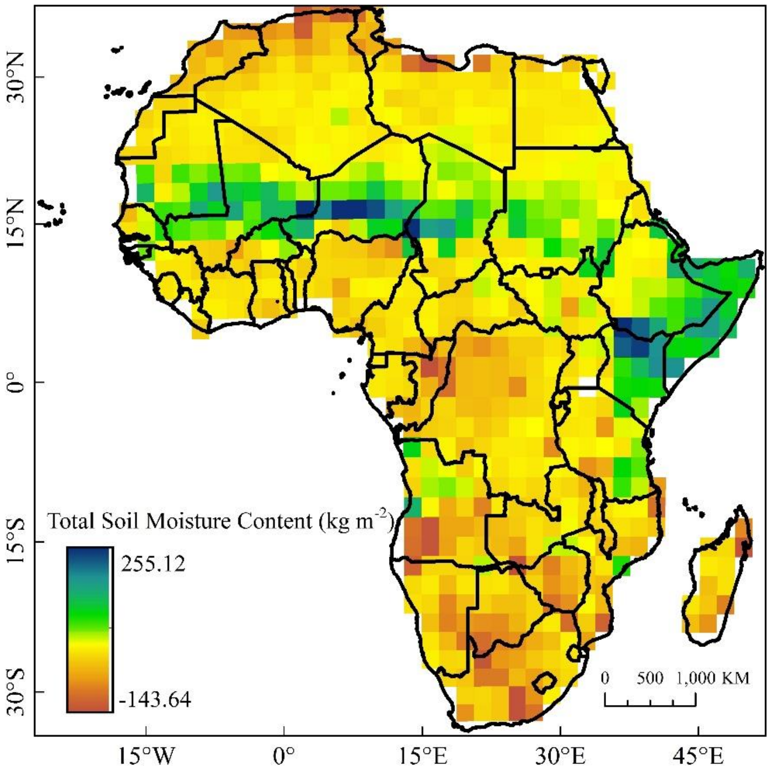

Supplementary Figure. 16 | Spatial distribution of good-quality observations within individual pixels of Landsat 4/5/7/8 images in 10 periods from 1984 to 2021 in Africa. The ten periods have durations of 3, 5, or 7 years. Due to a severe shortage of imagery, the 1991–1995 period was excluded from wetland classification and subsequent analyses.

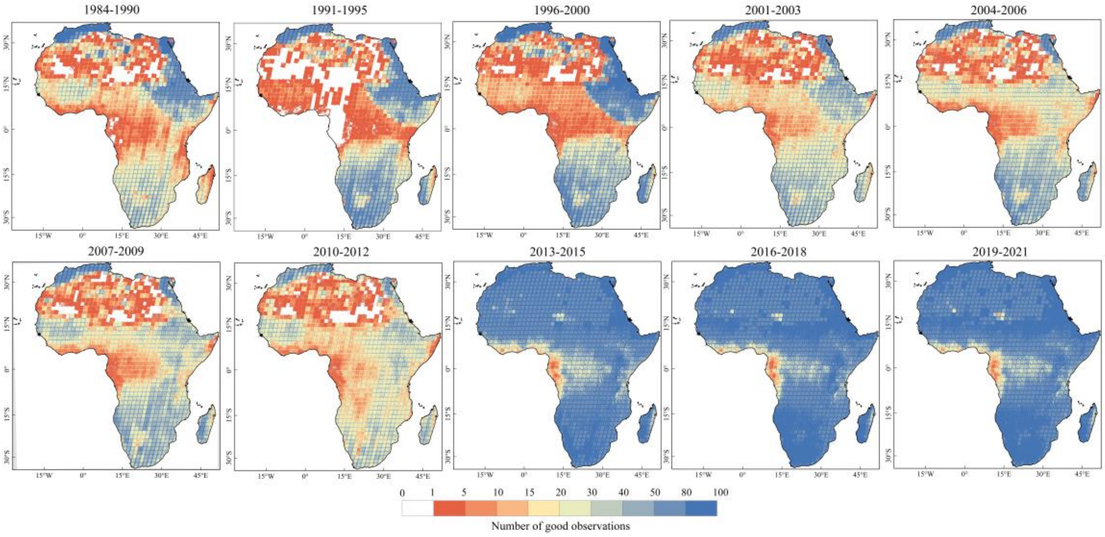

Supplementary Figure. 17 | Distribution of random validation points used for the wetland map consistency assessment. The wetland map for comparison was the RFW dataset, and the green validation points are those that were consistent with RFW, and the red ones are those that were not.

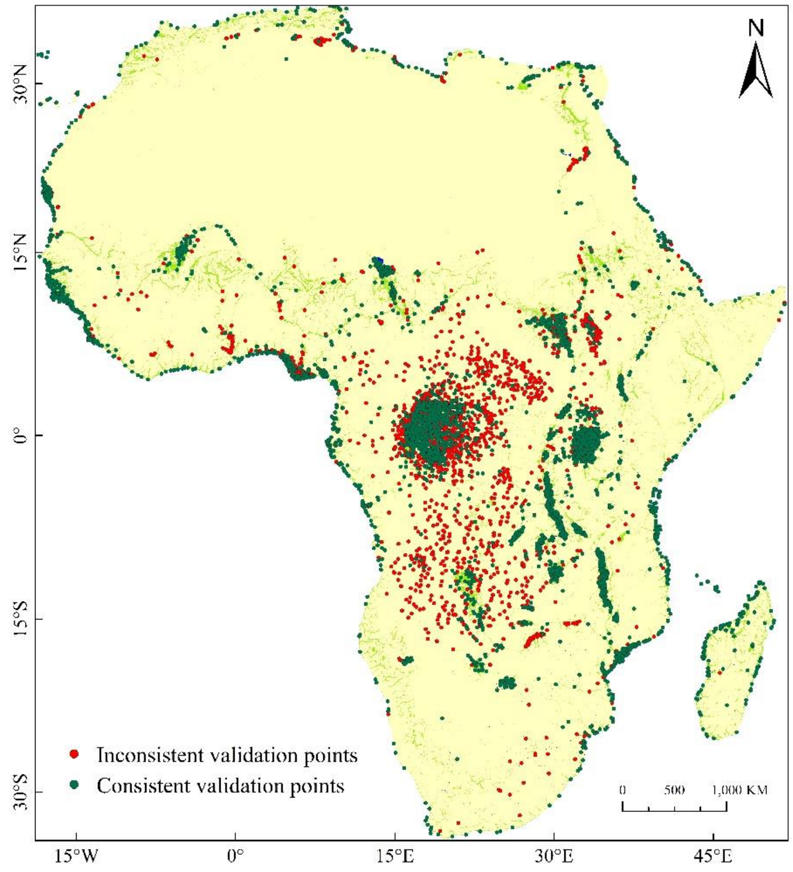

Supplementary Figure. 18 | The distribution of random verification points for the consistency assessment of specific wetland categories in Africa. (a) Banweulu Lake, inland marsh, distribution consistency of 82.14%; (b) Congo Basin, inland swamp, distribution consistency of 76.55%; (c) Coastal areas of Guinea-Bissau, coastal swamps, distribution consistency of 76.92%. The green verification point was consistent with the comparison map, and the red was inconsistent.

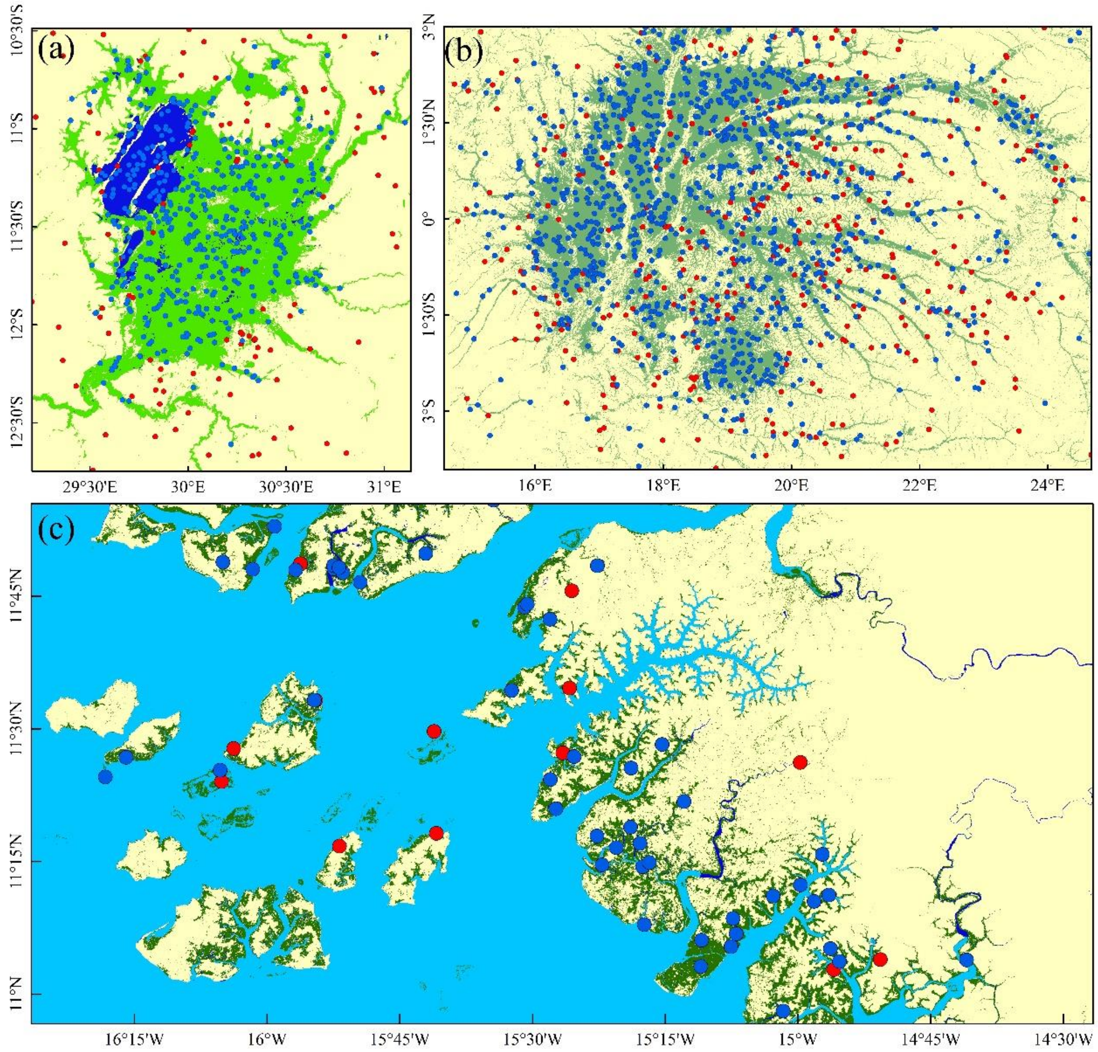

Supplementary Figure. 19 | Comparison of simulated wetland area time series and classified wetland area time series. The left coordinate represented the classified inland wetland area, and the right ordinate represented the simulated inland wetland area based on FLDAS soil moisture data. The vertical coordinates were the middle year of 10 periods.

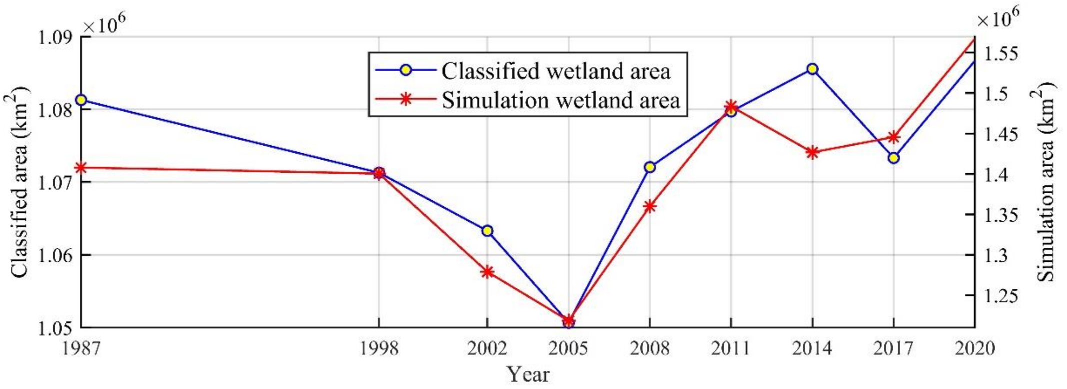

Supplementary Figure. 20 | Comparison of CMIP6 soil moisture time series and FLDAS soil moisture change trend. The FLDAS soil moisture represents the sum of soil moisture (kg/m²) across four layers: 0–10 cm, 10–40 cm, 40–100 cm, and 100–200 cm, reflecting the total amount of water contained in the soil per unit area. The CMIP6 soil moisture time series is derived by averaging the soil moisture data from 14 models.

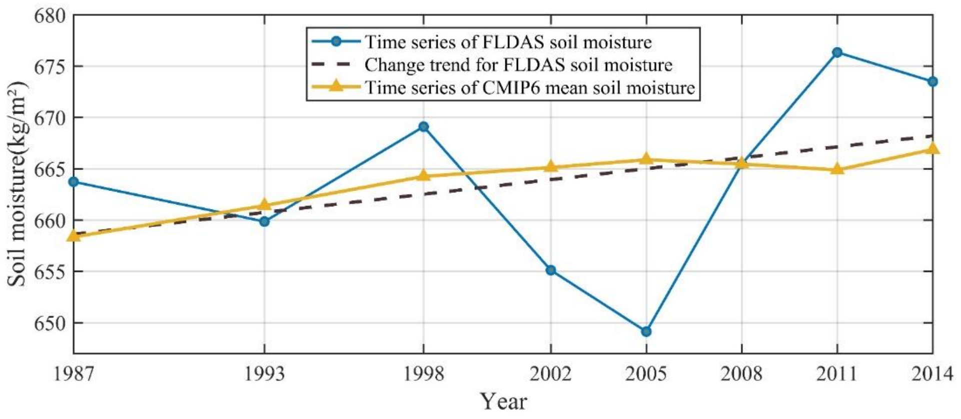

Supplementary Figure. 21 | Wetland extent analyzed at the subcontinental scale in Africa, with the background displaying the 2020 wetland classification results from this study. Wetland extent used for subcontinental-scale analysis of Africa. The background shows this study's 2020 wetland classification result.

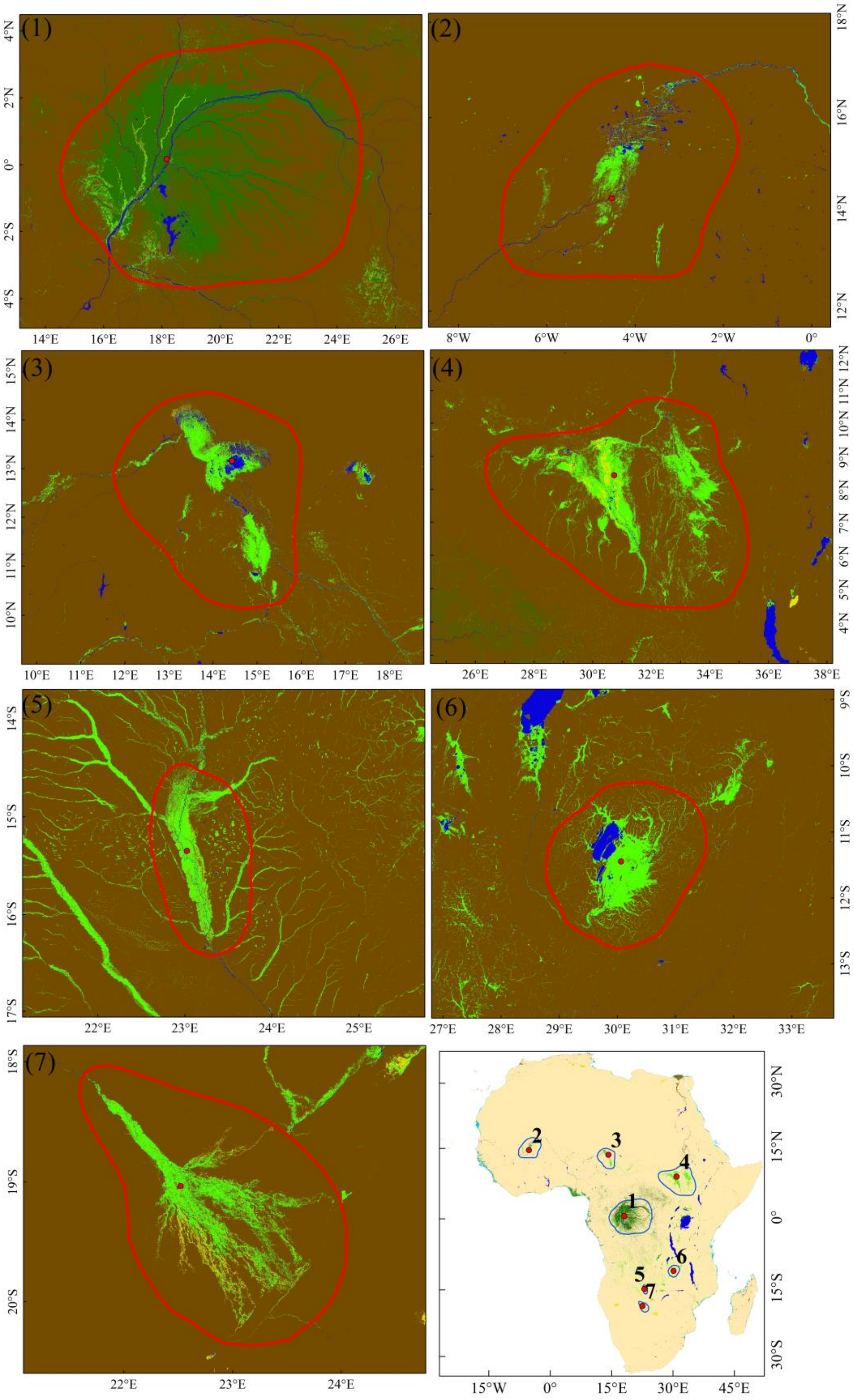

Supplementary Figure. 22 | Scatter charts comparing the current results against the African wetland flood extent from GIEMS-MC. Missing years in the study dataset were supplemented by linear interpolation. The trend line represents a linear fit of GIEMS-MC data from 2000 to 2020. All the statistical tests are two-sided.

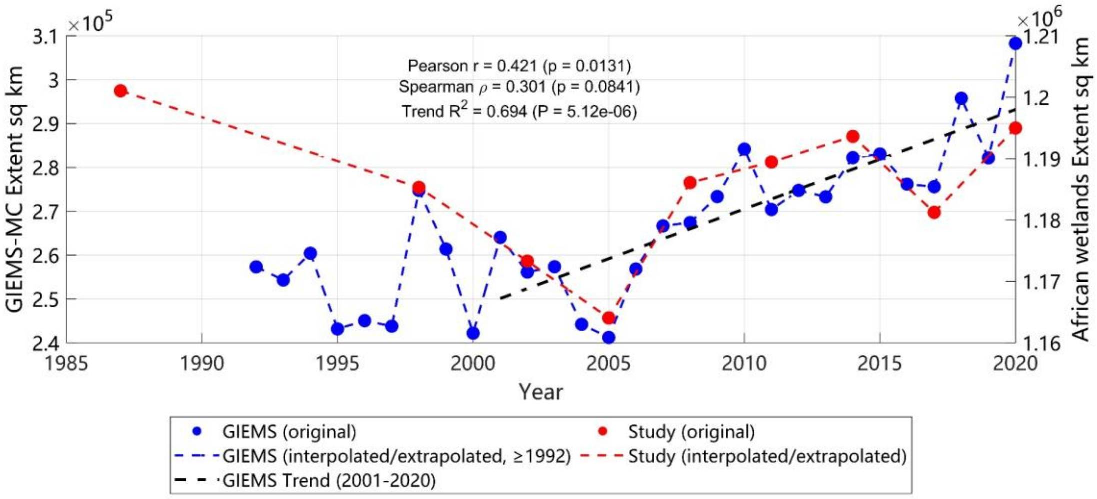

Supplementary Figure. 23 | Scatter charts comparing the current results against the African wetland flood extent from GWL_FCS30. Trend lines are obtained by one-dimensional linear fitting, and trend significance is assessed using the Mann–Kendall nonparametric trend test; the test statistic Z and two-sided P-values are reported (two-sided test).

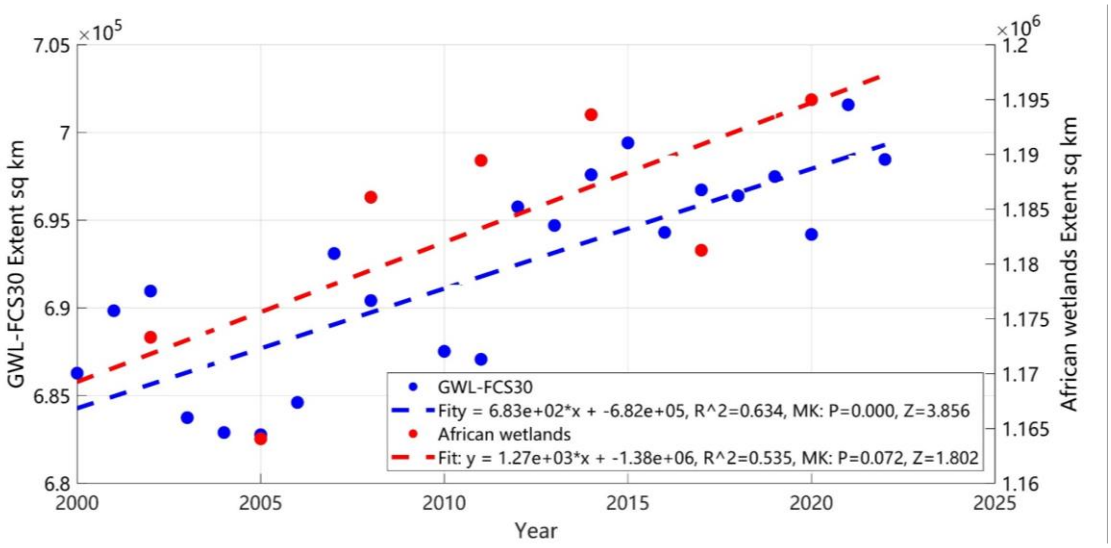

Supplementary Figure. 24 | Scatter charts comparing the current results against the African wetland flood extent from WAD2M. Trend lines are obtained by one-dimensional linear fitting, and trend significance is assessed using the Mann－Kendall nonparametric trend test; the test statistic Z and two-sided P-values are reported (two-sided test).

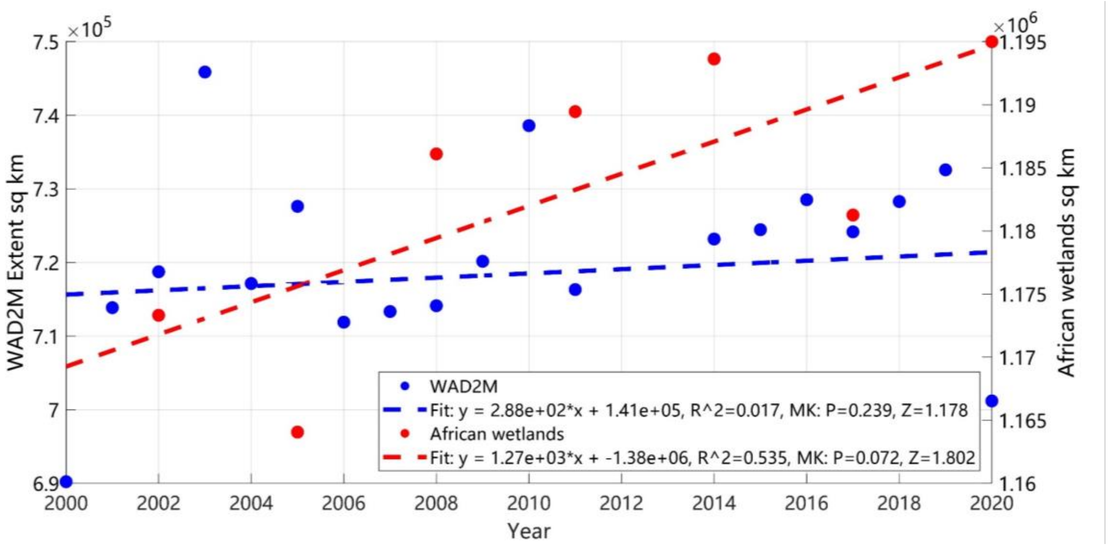

Supplementary Figure. 25 | Time series of projected temperature changes in Africa under different SSP scenarios. Gray lines show results from the 14 models; the colored line shows the mean across the 14 models.

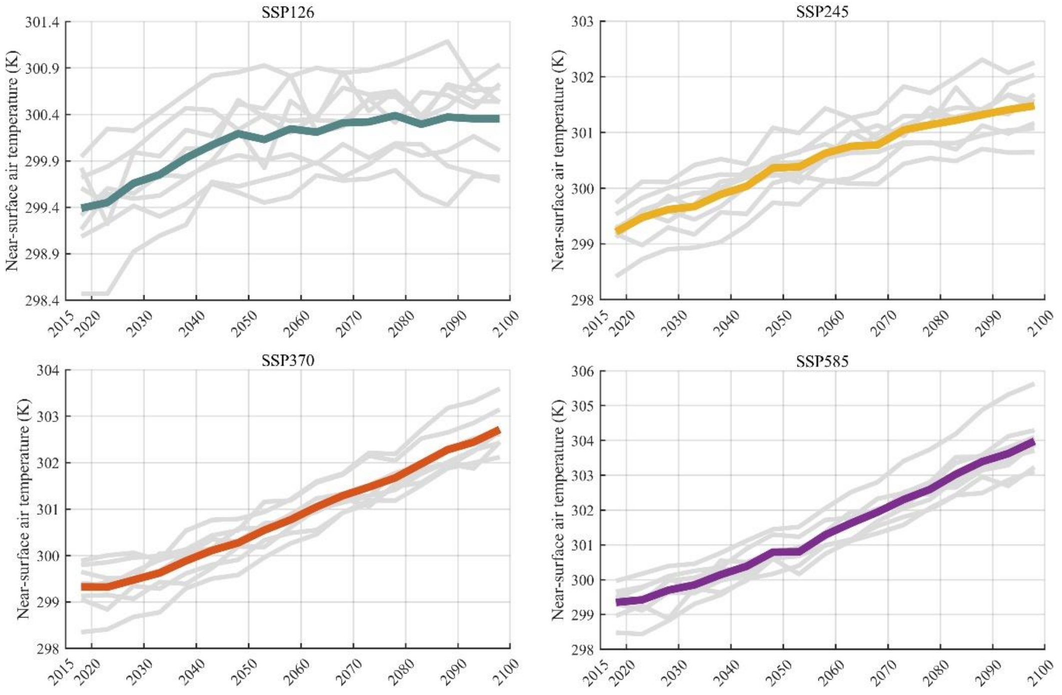

Supplementary Figure. 26 | Time series of projected precipitation changes in Africa under different SSP scenarios. Gray lines show results from the 14 models; the colored line shows the mean across the 14 models.

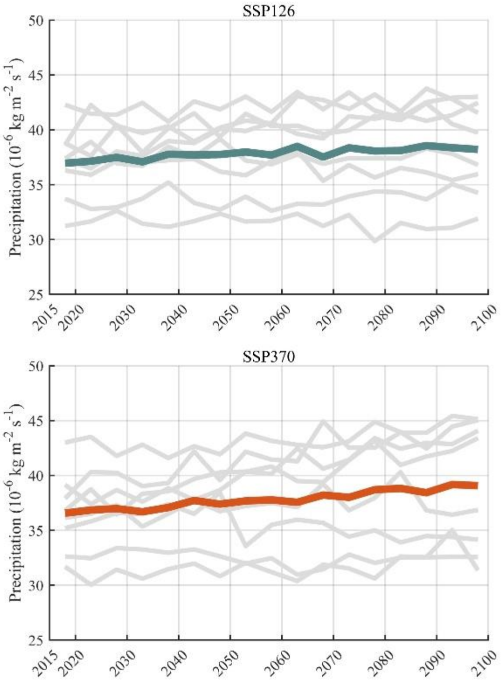

Supplementary Figure. 27 | Time series of projected soil moisture changes in Africa under different SSP scenarios. Gray lines show results from the 14 models; the colored line shows the mean across the 14 models.

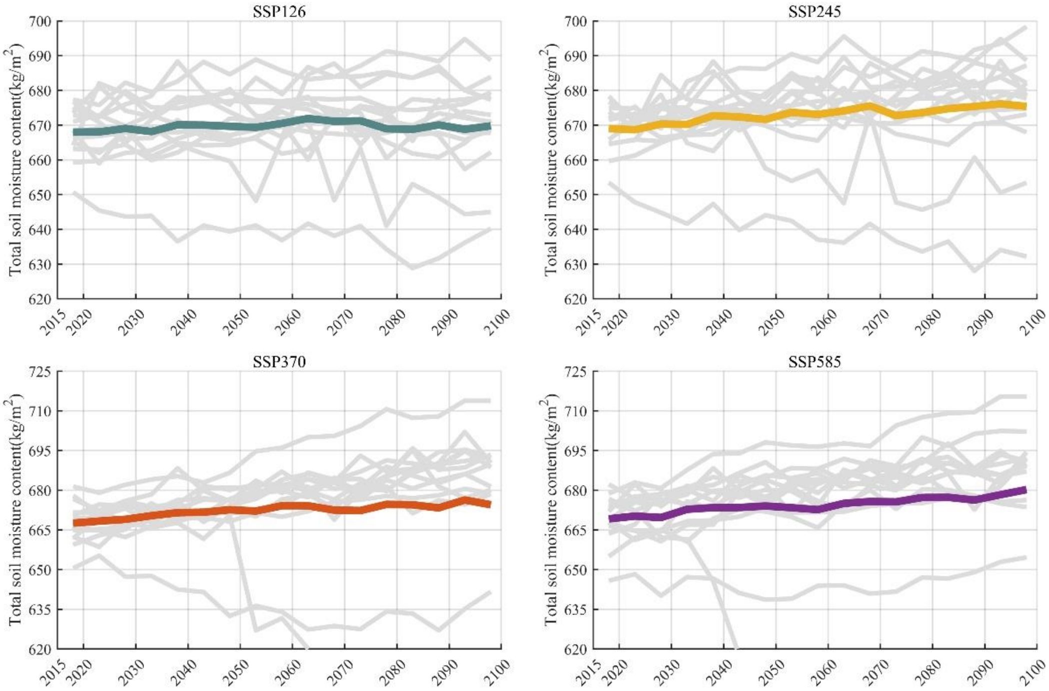

Supplementary References

1 Li, A. et al. Mapping African wetlands for 2020 using multiple spectral, geo-ecological features and Google Earth Engine. ISPRS J. Photogramm. Remote Sens. 193, 252-268 (2022).

2 Mahdavi, S. et al. Remote sensing for wetland classification: a comprehensive review. GISci. Remote Sens. 55, 623-658, doi:10.1080/15481603.2017.1419602 (2017).

3 Lehner, B. & Döll, P. Development and validation of a global database of lakes, reservoirs and wetlands. J. Hydrol. 296, 1-22 (2004).

4 Pekel, J.-F., Cottam, A., Gorelick, N. & Belward, A. S. High-resolution mapping of global surface water and its long-term changes. Nature 540, 418-422 (2016).

5 Gumbricht, T. et al. An expert system model for mapping tropical wetlands and peatlands reveals South America as the largest contributor. Global Change Biol. 23, 3581-3599 (2017).

6 Prigent, C., Jimenez, C. & Bousquet, P. Satellite‐derived global surface water extent and dynamics over the last 25 years (GIEMS‐2). J. Geophys. Res.: Atmos. 125, e2019JD030711 (2020).

7 Zhang, Z. et al. Development of a global dataset of Wetland Area and Dynamics for Methane Modeling (WAD2M). Earth Syst. Sci. Data Discuss. 2020, 1-50 (2020).

8 Prigent, C., Papa, F., Aires, F., Rossow, W. B. & Matthews, E. Global inundation dynamics inferred from multiple satellite observations, 1993–2000. J. Geophys. Res. 112, doi:10.1029/2006jd007847 (2007).

9 Tootchi, A., Jost, A. & Ducharne, A. Multi-source global wetland maps combining surface water imagery and groundwater constraints. Earth Syst. Sci. Data 11, 189-220, doi:10.5194/essd-11-189-2019 (2019).

10 Papa, F. et al. Interannual variability of surface water extent at the global scale, 1993–2004. J. Geophys. Res.: Atmos. 115 (2010).

11 Spalding, M. World atlas of mangroves. (Routledge, 2010).

12 Giri, C. et al. Status and distribution of mangrove forests of the world using earth observation satellite data. Global Ecol. Biogeogr. 20, 154-159, doi:10.1111/j.1466-8238.2010.00584.x (2011).

13 McOwen, C. J. et al. A global map of saltmarshes. Biodivers Data J, e11764, doi:10.3897/BDJ.5.e11764 (2017).

14 Murray, N. J. et al. The global distribution and trajectory of tidal flats. Nature 565, 222-225, doi:10.1038/s41586-018-0805-8 (2019).

15 Bartholome, E. & Belward, A. S. GLC2000: a new approach to global land cover mapping from Earth observation data. Int. J. Remote Sens. 26, 1959-1977 (2005).

16 Arino O. et al. Global land cover map for 2009 (GlobCover 2009). PANGAEA, doi: 10.1594/PANGAEA.787668 (2012).

17 Friedl, M. A. et al. MODIS Collection 5 global land cover: Algorithm refinements and characterization of new datasets. Remote Sens. Environ. 114, 168-182 (2010).

18 Chen, J. et al. Global land cover mapping at 30m resolution: A POK-based operational approach. ISPRS J. Photogramm. Remote Sens. 103, 7-27, doi:10.1016/j.isprsjprs.2014.09.002 (2015).

19 Adams, J. B. Salt marsh at the tip of Africa: Patterns, processes and changes in response to climate change. Estuarine Coastal Shelf Sci. 237, doi:10.1016/j.ecss.2020.106650 (2020).

20 Jacob, A. L., Bonnell, T. R., Dowhaniuk, N. & Hartter, J. Topographic and spectral data resolve land cover misclassification to distinguish and monitor wetlands in western Uganda. ISPRS J. Photogramm. Remote Sens. 94, 114-126, doi:10.1016/j.isprsjprs.2014.05.001 (2014).
21 Bwangoy, J.-R. B., Hansen, M. C., Roy, D. P., Grandi, G. D. & Justice, C. O. Wetland mapping in the Congo Basin using optical and radar remotely sensed data and derived topographical indices. Remote Sens. Environ. 114, 73-86, doi:10.1016/j.rse.2009.08.004 (2010).
22 Schuerch, M. et al. Future response of global coastal wetlands to sea-level rise. Nature 561, 231-234 (2018).
23 Xi, Y., Peng, S., Ciais, P. & Chen, Y. Future impacts of climate change on inland Ramsar wetlands. Nat. Clim. Change 11, 45-51 (2021).
24 Fluet-Chouinard, E. et al. Extensive global wetland loss over the past three centuries. Nature 614, 281-286 (2023).
25 Eyring, V. et al. Overview of the Coupled Model Intercomparison Project Phase 6 (CMIP6) experimental design and organization. Geosci. Model Dev. 9, 1937-1958 (2016).
26 Babaousmail, H. et al. Evaluation of the performance of CMIP6 models in reproducing rainfall patterns over North Africa. Atmosphere 12, 475 (2021).
27 Dosio, A. et al. Projected future daily characteristics of African precipitation based on global (CMIP5, CMIP6) and regional (CORDEX, CORDEX-CORE) climate models. Clim. Dyn. 57, 3135-3158 (2021).
28 Dosio, A., Lennard, C. & Spinoni, J. Projections of indices of daily temperature and precipitation based on bias-adjusted CORDEX-Africa regional climate model simulations. Clim. Change 170, 13 (2022).
29 Bobde, V., Akinsanola, A., Folorunsho, A., Adebiyi, A. & Adeyeri, O. Projected regional changes in mean and extreme precipitation over Africa in CMIP6 models. Environ. Res. Lett. (2024).
30 Almazroui, M. et al. Projected change in temperature and precipitation over Africa from CMIP6. Earth Syst. Environ. 4, 455-475 (2020).
31 Lim Kam Sian, K. T. C., Wang, J., Ayugi, B. O., Nooni, I. K. & Ongoma, V. Multi-decadal variability and future changes in precipitation over Southern Africa. Atmosphere 12, 742 (2021).
32 Ibrahim, B. et al. Identification of a Representative Stationary Period for Rainfall Variability Description in the Sudano-Sahelian Zone of West Africa during the 1901 – 2018 Period. Atmosphere 12, 716 (2021).
33 Tucker, C. J. Red and photographic infrared linear combinations for monitoring vegetation. Remote Sens. Environ. 8, 127-150 (1979).
34 Huete, A., Liu, H., Batchily, K. & Van Leeuwen, W. A comparison of vegetation indices over a global set of TM images for EOS-MODIS. Remote Sens. Environ. 59, 440-451 (1997).
35 McFeeters, S. K. The use of the Normalized Difference Water Index (NDWI) in the delineation of open water features. Int. J. Remote Sens. 17, 1425-1432 (1996).
36 Xu, H. Modification of normalised difference water index (NDWI) to enhance open water features in remotely sensed imagery. Int. J. Remote Sens. 27, 3025-3033 (2006).
37 Tiner, R. W., Lang, M. W. & Klemas, V. V. Remote sensing of wetlands: applications and advances. (CRC press, 2015).
38 Wood, J. The geomorphological characterisation of digital elevation models. (University of

Leicester (United Kingdom), 1996).
39 MacMillan, R., Pettapiece, W., Nolan, S. & Goddard, T. A generic procedure for automatically segmenting landforms into landform elements using DEMs, heuristic rules and fuzzy logic. Fuzzy Sets Syst. 113, 81-109 (2000).
40 McNally, A. et al. A land data assimilation system for sub-Saharan Africa food and water security applications. Sci. Data 4, 1-19 (2017).
41 Cook, B. I. et al. Twenty - first century drought projections in the CMIP6 forcing scenarios. Earth's Future 8, e2019EF001461 (2020).
42 Lehner, B., Verdin, K. & Jarvis, A. New global hydrography derived from spaceborne elevation data. Eos, Transactions American Geophysical Union 89, 93-94 (2008).
43 Marthews, T., Dadson, S., Lehner, B., Abele, S. & Gedney, N. High-resolution global topographic index values for use in large-scale hydrological modelling. Hydrol. Earth Syst. Sci. 19, 91-104 (2015).
44 Zhang, Z., Zimmermann, N. E., Kaplan, J. O. & Poulter, B. Modeling spatiotemporal dynamics of global wetlands: comprehensive evaluation of a new sub-grid TOPMODEL parameterization and uncertainties. Biogeosciences 13, 1387-1408 (2016).
45 Stocker, B., Spahni, R. & Joos, F. DYPTOP: a cost-efficient TOPMODEL implementation to simulate sub-grid spatio-temporal dynamics of global wetlands and peatlands. Geosci. Model Dev. 7, 3089-3110 (2014).
46 Mu, H. et al. A global record of annual terrestrial Human Footprint dataset from 2000 to 2018. Sci. Data 9, 176 (2022).
