# Supplementary material for: African inland wetland area on the rise during the 21st century
Source: Nat Commun. 2026 Mar 6;17:3600. doi: 10.1038/s41467-026-70480-6 (PMC13096438; doi:10.1038/s41467-026-70480-6)
Supplement: Supplementary file 2 — Reporting Summary [file 41467_2026_70480_MOESM2_ESM.pdf]

nature portfolio

Corresponding author(s): Shengbo Chen, Yuanzhi Zhan

Last updated by author(s): Dec 17, 2025

Reporting Summary

Nature Portfolio wishes to improve the reproducibility of the work that we publish. This form provides structure for consistency and transparency in reporting. For further information on Nature Portfolio policies, see our Editorial Policies and the Editorial Policy Checklist.

Statistics

For all statistical analyses, confirm that the following items are present in the figure legend, table legend, main text, or Methods section.

n/a	Confirmed	
☐	☒	The exact sample size (*n*) for each experimental group/condition, given as a discrete number and unit of measurement
☐	☒	A statement on whether measurements were taken from distinct samples or whether the same sample was measured repeatedly
☐	☒	The statistical test(s) used AND whether they are one- or two-sided<br>*Only common tests should be described solely by name; describe more complex techniques in the Methods section.*
☐	☒	A description of all covariates tested
☐	☒	A description of any assumptions or corrections, such as tests of normality and adjustment for multiple comparisons
☐	☒	A full description of the statistical parameters including central tendency (e.g. means) or other basic estimates (e.g. regression coefficient) AND variation (e.g. standard deviation) or associated estimates of uncertainty (e.g. confidence intervals)
☐	☒	For null hypothesis testing, the test statistic (e.g. *F*, *t*, *r*) with confidence intervals, effect sizes, degrees of freedom and *P* value noted<br>*Give P values as exact values whenever suitable.*
☒	☐	For Bayesian analysis, information on the choice of priors and Markov chain Monte Carlo settings
☒	☐	For hierarchical and complex designs, identification of the appropriate level for tests and full reporting of outcomes
☐	☒	Estimates of effect sizes (e.g. Cohen's *d*, Pearson's *r*), indicating how they were calculated

Our web collection on statistics for biologists contains articles on many of the points above.

Software and code

Policy information about availability of computer code

Data collection	All data used in this study is available for online download, with no use of specific code or software during data collection.
Data analysis	The random forest algorithm implemented in Google Earth Engine was developed by Leo Breiman (https://rd.springer.com/article/10.1023/A:1010933404324).<br>The TOPMODEL method described by Yi Xi was adapted to JavaScript code for implementation in Google Earth Engine (https://doi.org/10.1038/s41558-020-00942-2).<br>Spatial analysis and validation of map data were conducted in Google Earth Engine, and data analyses were performed using the ggplot2, trend, Kendall, and caret packages in R version 4.4.1 (https://cran.r-project.org).<br>Data visualization was accomplished using R v.4.4.1. and QGIS Desktop 3.34.1.<br>The code used for data analysis is available at zenodo (https://doi.org/10.5281/zenodo.17865977).

For manuscripts utilizing custom algorithms or software that are central to the research but not yet described in published literature, software must be made available to editors and reviewers. We strongly encourage code deposition in a community repository (e.g. GitHub). See the Nature Portfolio guidelines for submitting code & software for further information.

Data

Policy information about availability of data

All manuscripts must include a data availability statement. This statement should provide the following information, where applicable:

- Accession codes, unique identifiers, or web links for publicly available datasets
- A description of any restrictions on data availability
- For clinical datasets or third party data, please ensure that the statement adheres to our policy

All data used in this study are freely available from public repositories. The Landsat images used in this study are available from the US Geological Survey (http://earthexplorer.usgs.gov) and Google Earth Engine (https:// earthengine.google.com). The data of African national boundaries and 6-meter water depth boundaries are available from https://developers.google.com/earth-engine/datasets/catalog/USDOS_LSIB_SIMPLE_2017 and https://developers.google.com/earth-engine/datasets/catalog/NOAA_NGDC_ETOPO1. Human Impact Index (HII) data is available from the Wildlife Conservation Society�WCS� (https://wcshumanfootprint.org/data-access). Temperature data are available from https://developers.google.com/earth-engine/datasets/catalog/ECMWF_ERA5_MONTHLY. Precipitation data are available from https://developers.google.com/earth-engine/datasets/catalog/IDAHO_EPSCOR_TERRACLIMATE, https://developers.google.com/earth-engine/datasets/catalog/NASA_FLDAS_NOAH01_C_GL_M_V001 , and https://www.ncei.noaa.gov/data/global-precipitation-climatology-project-gpcp-monthly/access/. PDSI is available from https://developers.google.com/earth-engine/datasets/catalog/IDAHO_EPSCOR_TERRACLIMATE. Soil moisture is available from https://developers.google.com/earth-engine/datasets/catalog/NASA_FLDAS_NOAH01_C_GL_M_V001. CTI data is available from https://catalogue.ceh.ac.uk/documents/6b0c4358-2bf3-4924-aa8f-793d468b92be. Africa watershed vector data is available from https://developers.google.com/earth-engine/datasets/catalog/WWF_HydroSHEDS_v1_Basins_hybas_8. CMIP6 data is available from https://esgf-node.llnl.gov/search/cmip6/.
The wetland maps for ten historical periods and the wetland simulation results for future periods produced in this study have been deposited in the Zenodo database and are provided as open data�https://doi.org/10.5281/zenodo.17865977�.

Research involving human participants, their data, or biological material

Policy information about studies with human participants or human data. See also policy information about sex, gender (identity/presentation), and sexual orientation and race, ethnicity and racism.

Reporting on sex and gender	This study did not involve human participants, their data, or biological material.
Reporting on race, ethnicity, or other socially relevant groupings	This study did not involve human participants, their data, or biological material.
Population characteristics	This study did not involve human participants, their data, or biological material.
Recruitment	This study did not involve human participants, their data, or biological material.
Ethics oversight	This study did not involve human participants, their data, or biological material.

Note that full information on the approval of the study protocol must also be provided in the manuscript.

Field-specific reporting

Please select the one below that is the best fit for your research. If you are not sure, read the appropriate sections before making your selection.

☐ Life sciences ☐ Behavioural & social sciences ☒ Ecological, evolutionary & environmental sciences

For a reference copy of the document with all sections, see nature.com/documents/nr-reporting-summary-flat.pdf

Ecological, evolutionary & environmental sciences study design

All studies must disclose on these points even when the disclosure is negative.

Study description	This study mapped African wetlands and provided an assessment of area loss, drivers, and future trends under climate change in African with 270,000 sampling points, 810,000 Landsat images, and soil moisture data from 14 CMIP6 models.
Research sample	Analytical samples comprise ~270,000 visually interpreted reference points and ~810,000 Landsat scenes (1984–2021) used to produce nine epochal 30-m wetland maps. Auxiliary datasets: ERA5 (temperature), TerraClimate (PDSI), FLDAS (historical SM), CMIP6 SM (future), Human Impact Index (HII), hydrobasins/CTI.
Sampling strategy	Points were placed systematically on a 0.1° grid and visually interpreted against multi-temporal composites; 70% of points were used for training and 30% for independent validation. No formal power calculation was applied because the task is continent-scale classification; sample size was chosen to maximize spatial and class coverage and is supported by classification accuracy.
Data collection	Trained interpreters labeled points within Google Earth Engine (GEE). Preprocessing, feature extraction and Random Forest classification were executed by reproducible GEE scripts; downstream analyses and TOPMODEL runs used standard scientific

	computing environments. All scripts and metadata were archived. Details for the data collection procedure were described in the Methods section.
Timing and spatial scale	Historical mapping: 1984–2021 (nine epochs at 30-m). Future simulation: 2015–2100 (CMIP6 SSP scenarios). Spatial extent: entire African continent and affiliated islands; sampling grid ~0.1°; analysis resolution 30 m; TOPMODEL applied to ~40,000 basins.
Data exclusions	No data were excluded from the analysis.
Reproducibility	All source datasets are public; preprocessing, classification and analysis scripts were implemented as reproducible GEE scripts and standard code (archived). TOPMODEL parameter calibration and RMSE diagnostics are described in Methods. Final wetland maps and simulation outputs have been deposited with metadata
Randomization	Randomization applied where relevant: interpreted points were randomly partitioned into training (70%) and independent validation (30%). Spatial subregion assignment was non-random for computational reasons; potential covariate effects were mitigated by stratified sampling, inclusion of multi-temporal and auxiliary predictors, and regional calibration procedures.
Blinding	This study does not involve blinding, as all data are derived from observations or reanalysis and are not influenced by expectations or prior knowledge.

Did the study involve field work? ☐ Yes ☒ No

Reporting for specific materials, systems and methods

We require information from authors about some types of materials, experimental systems and methods used in many studies. Here, indicate whether each material, system or method listed is relevant to your study. If you are not sure if a list item applies to your research, read the appropriate section before selecting a response.

Materials & experimental systems

n/a	Involved in the study
☒	☐ Antibodies
☒	☐ Eukaryotic cell lines
☒	☐ Palaeontology and archaeology
☒	☐ Animals and other organisms
☒	☐ Clinical data
☒	☐ Dual use research of concern
☒	☐ Plants

Methods

n/a	Involved in the study
☒	☐ ChIP-seq
☒	☐ Flow cytometry
☒	☐ MRI-based neuroimaging

Plants

Seed stocks	This study did not involve plants
Novel plant genotypes	This study did not involve plants
Authentication	This study did not involve plants
